# State-Dependent Resource Harvesting with Lagged Information about System States

**DOI:** 10.1371/journal.pone.0157373

**Published:** 2016-06-17

**Authors:** Fred A. Johnson, Paul L. Fackler, G. Scott Boomer, Guthrie S. Zimmerman, Byron K. Williams, James D. Nichols, Robert M. Dorazio

**Affiliations:** 1 Wetland and Aquatic Research Center, U. S. Geological Survey, Gainesville, Florida, United States of America; 2 Department of Agriculture and Resource Economics, North Carolina State University, Raleigh, North Carolina, United States of America; 3 Division of Migratory Bird Management, U. S. Fish and Wildlife Service, Laurel, Maryland, United States of America; 4 Division of Migratory Bird Management, U.S. Fish and Wildlife Service, Sacramento, California, United States of America; 5 The Wildlife Society, Bethesda, Maryland, United States of America; 6 Patuxent Wildlife Research Center, U.S. Geological Survey, Laurel, Maryland, United States of America; US Army Engineer Research and Development Center, UNITED STATES

## Abstract

Markov decision processes (MDPs), which involve a temporal sequence of actions conditioned on the state of the managed system, are increasingly being applied in natural resource management. This study focuses on the modification of a traditional MDP to account for those cases in which an action must be chosen after a significant time lag in observing system state, but just prior to a new observation. In order to calculate an optimal decision policy under these conditions, possible actions must be conditioned on the previous observed system state and action taken. We show how to solve these problems when the state transition structure is known and when it is uncertain. Our focus is on the latter case, and we show how actions must be conditioned not only on the previous system state and action, but on the probabilities associated with alternative models of system dynamics. To demonstrate this framework, we calculated and simulated optimal, adaptive policies for MDPs with lagged states for the problem of deciding annual harvest regulations for mallards (*Anas platyrhynchos*) in the United States. In this particular example, changes in harvest policy induced by the use of lagged information about system state were sufficient to maintain expected management performance (e.g. population size, harvest) even in the face of an uncertain system state at the time of a decision.

## Introduction

There is a growing literature concerned with the application of optimization methods [1–3] to dynamic decision-making problems in conservation [4–12]. Dynamic decision problems are common in natural resources applications, including the management of habitats, the control of invasive species, and the stocking, transplanting, or harvesting of organisms. The growing number of applications of dynamic optimization methods is testament to their broad applicability, and rapid increases in computing resources has made it feasible to analyze problems of at least moderate complexity [13].

Dynamic optimization methods combine models of system change with objective functions that calculate the value of alternative management actions over time [13]. A common approach in resource management is to characterize a problem as a Markov decision process, which involves a temporal sequence of decisions, with policies that identify actions at each decision point depending on time and system state [1]. The goal of the manager is to develop a decision rule (or management policy) that prescribes management actions for each time and system state that maximizes (or minimizes) the temporal sum of objective values. A key advantage when optimizing Markov decision processes is the ability to produce a feedback (or closed-loop) policy specifying optimal decisions for *possible* future system states rather than *expected* future states [2]. In practice this makes dynamic optimization appropriate for systems that behave stochastically, absent any assumptions about the system remaining in a desired equilibrium or about the production of a constant stream of resource returns.

A key consideration in a dynamic framing of natural resource problems is the uncertainty attendant to management outcomes. In addition to demographic and environmental variation, uncertainty about resource changes may arise from errors in measurement and sampling of ecological systems (partial system observability), incomplete control of management actions (partial control), and incomplete knowledge of system behavior (structural uncertainty) [14]. A failure to recognize and account for these uncertainties can depress management performance and lead to environmental and economic losses [15]. Thus, there is increasing emphasis on methods that can account for uncertainty about the dynamics of ecological systems and their uncertain responses to both controlled and uncontrolled factors [16–18].

Adaptive management is an approach for coping with structural uncertainty by explicitly accounting for multiple, competing models of the system’s dynamics [16, 19–23]. We emphasize that the term “adaptive management” as we use it here deals specifically with the issue of structural uncertainty in a repeated decision context; we acknowledge that the term is often used more broadly to encompass many other aspects of collaborative decision making under uncertainty [24–31]. Structural uncertainty in system dynamics can often be expressed as alternative models, characterized by continuous or discrete probability distributions of model parameters, or by alternative model forms that are hypothesized or estimated from historic data. The notion of adaptation arises from the recognition that these probability distributions are not static, but evolve over time as new observations of system behaviors are accumulated from the management process. Indeed, the defining characteristic of adaptive management is the attempt to account for the temporal dynamics of this uncertainty in making management decisions [16, 17, 20–22, 32]. Stochastic control methods [3, 13], with their focus on dynamic decisions and the uncertainties attendant to future outcomes, are particularly well suited for formulating adaptive management policies. An increasing number of examples of passive and active adaptive management policies can be found in the conservation literature [33–36]. Both passive and active adaptive management treat probabilities of the alternative parameters or models as part of the system state, but only active adaptive management explicitly incorporates the potential for learning in assessing and selecting management actions [13].

There has been growing attention to the problem of partial system observability, as it presents a common challenge in conservation because system state is almost never known with certainty [37–42]. Our concern here is with the adaptive management of resource harvesting, subject to partial system observability. Partial observability often stems from sampling or measurement error in monitoring programs, but here we focus on additional uncertainty associated with estimation of system state prior to the time at which the action is to be taken, effectively necessitating projection of system state from the time of monitoring to the time of action. This particular perspective was motivated by a Supplemental Environmental Impact Statement (SEIS) issued by the United States Fish and Wildlife Service, concerning the timing of the annual process for regulating the harvests of migratory birds [43]. The modeling of the current decision process is naturally based on a specific temporal sequence of process events (the monitoring, decision and action), and a change in this timing necessitates an examination of potential changes to the corresponding optimization. Specifically, the implementation of the SEIS protocol increases the time lag between the monitoring on which the decision is based and the time at which the action is to be taken. We can envision other such situations in which a decision must be made at a time that does not immediately follow the acquisition of monitoring data, perhaps for administrative or legal reasons. To accommodate these cases there must be a modification of the classic formulation of Markov decision processes in which decisions are made immediately or soon after system state is assessed. This typically means that a new decision must be made prior to observing the effects of the last decision (and other uncontrollable factors) on system state. In order to calculate an optimal policy under these conditions, the system state must be augmented by a variable indicating the last decision made (i.e., the specific action taken). Decisions are thus conditioned on a time-lagged assessment of system state. Although this complicates the computation of optimal policies, it does not violate the Markovian property of the decision process.

Although our analyses were motivated by a specific case dealing with proposed changes in timing of decisions related to migratory bird harvest, such issues of decision timing have arisen in other decision processes as well. An adaptive management program has been adopted by the Atlantic States Marine Fishery Commission for the establishment of horseshoe crab harvest quotas in Delaware Bay [44]. State variables relevant to harvest decisions include not only the abundance of the harvested species, but also that of migratory shorebirds (red knots, *Calidris canutus*) that depend on eggs of horseshoe crabs as a food source at key migration stopover sites in Delaware Bay. Harvest quotas for the fishing season of June-December, year *t*+1, are established in the fall (e.g., November) of year *t*. The decision is informed by estimates of system-state variables obtained in May of year *t* (red knots) and October-November of year *t*-1 (horseshoe crabs). Debate has ensued about the feasibility of pushing the harvest decision forward (e.g., January, year *t*+1) to make use of the previous fall’s crab survey data.

Our objectives here are to describe a dynamic-optimization framework that accounts for lack of monitoring information on the current system state at the time of decision making, while also accounting for structural uncertainty in system dynamics. We demonstrate the framework using the regulation of mallard (*Anas platyrhynchos*) harvests in the United States as a case study.

## Methods

### Markov decision processes

We begin by describing a standard discrete-time infinite-horizon Markov decision process [45–47]. The resource system is characterized by a system state *x*_*t*_ at each time *t*, which represents key resource elements, features, and attributes that evolve through time. Examples include population size or density, structural features of habitats, or extant environmental conditions. We assume for now that the state of the system can be observed at the time a decision is made, and that structural components of the system that influence dynamics are known.

As described in detail elsewhere [13, 48, 49], the management process consists of actions *a*_*t*_ chosen at each time *t* from a set of options that are available at that time. A policy *A* prescribes actions to be taken at time *t*, so *a*_*t*_ = *A*(*x*_*t*_). System dynamics are assumed to be Markovian, in that the system state at time *t*+1 is determined stochastically by the state at time *t* and action taken at time *t*. These transitions are specified by a transition function *x*_*t*+1_ = *f*(*x*_*t*_, *a*_*t*_, *z*_*t*_), where the random variable *z*_*t*_ represents uncontrolled environmental variation that induces stochasticity in the transition function and implies a conditional probability distribution *P*(*x*_*t*+1_|*x*_*t*_, *a*_*t*_) for the transition from *x*_*t*_ to *x*_*t*+1_ assuming action *a*_*t*_ is taken.

Assuming the transition structure is known, a value function *V*(*x*_*t*_|*A*) captures the value of decisions made over time in terms of the model-based transition probabilities *P*(*x*_*t*+1_|*x*_*t*_, *a*_*t*_) and accumulated utilities *U*(*x*_*t*_, *a*_*t*_). This notation suggests that utility is influenced by the action *a*_*t*_ taken at time *t* as well as the system state *x*_*t*_ at that time. Dynamic decision making typically is based on an objective or value function that accumulates discounted expected utilities from the current time forward:

$$V\left(x_{t}|A\right)=E\left[\sum_{h=0}^{\infty }\lambda^{h}U\left(x_{t+h},A\left(x_{t+h}\right)\right)|x_{t},A\left(x_{t}\right)\right]$$ (1)

where the value *V*(*x*_*t*_|*A*) corresponding to policy *A* is conditional on the resource state *x*_*t*_ and the expectation is with respect to environmental variation and other measures of uncertainty. One way to characterize the relative importance of future vs. current values is to include a discount factor *λ* ≤ 1 for the time-specific utilities in Eq (1) that reduces future utility relative to current utility. The goal is to find the policy *A* that maximizes overall system utility *V*. Solution approaches typically make use of Bellman’s equation:

$$V\left(x_{t}|A\right)=U\left(x_{t},A\left(x_{t}\right)\right)+\lambda \sum_{x_{t+1}}P\left(x_{t+1}|x_{t},A\left(x_{t}\right)\right)V(x_{t+1}|A)$$ (2)

which can be used in iterative routines such as function and policy iteration [46]. When *λ* = 1 the value function as stated may not converge and the Bellman equation must be modified. This situation is generally associated with so-called ergodic control [46] that attempts to maximize the time-averaged utility rather than a temporal sum of utilities:

$$\underset{A\left(x_{t}\right)}{\text{argmax}}\underset{T\to \infty }{\text{lim}}\frac{1}{T}\sum_{t=1}^{T}E\left[U\left(x_{t},A\left(x_{t}\right)\right)\right].$$ (3)

If there is uncertainty about the transition structure, several candidate models can be used to describe state transitions, with *f*_*k*_ and *P*_*k*_(*x*_*t*+1_|*x*_*t*_, *a*_*t*_) representing a particular model *k* ∈ {1, 2,.., *K*}. Structural (or model) uncertainty can be characterized by a distribution *q*_*t*_ consisting of *K* model likelihoods or weights, with elements *q*_*t*_(*K*) that sum to one. Here we refer to the distribution of model weights as the model state to distinguish it from the system state *x*_*t*_. The transition probability for the system state *x* is now conditional on the current model state as well as the current system state and action and is given by:

$$P\left(x_{t+1}|x_{t},q_{t},a_{t}\right)=\sum_{k=1}^{K}q_{t}\left(k\right)P_{k}\left(x_{t+1}|x_{t},a_{t}\right).$$ (4)

The model state is updated once the next period’s state is observed using an updating rule *q*_*t*+1_ = *g*(*x*_*t*+1_, *x*_*t*_, *q*_*t*_, *a*_*t*_) that typically (but not necessarily) uses Bayes’ theorem, based on the relative likelihood of *x*_*t*+1_ under each of the alternative models.

When there is uncertainty about the transition structure, the utility function, value function and strategy all become functions of the model state *q*_*t*_. When the utility function depends on the model, we can write the utility function as:

$$U\left(x_{t},q_{t},a_{t}\right)=\sum_{k=1}^{K}q_{t}\left(k\right)U_{k}\left(x_{t},a_{t}\right)$$ (5)

The value function can then be expressed as:

$$V\left(x_{t},q_{t}|A\right)=E\left[\sum_{h=0}^{\infty }\lambda^{h}U\left(x_{t+h},q_{t+h},A\left(x_{t+h},q_{t+h}\right)\right)|x_{t},q_{t},A\left(x_{t},q_{t}\right)\right]$$ (6)

It is important to note that the strategy *A* is now a function of both system state *x* and model state *q*. Although this adds complexity, the problem is essentially the same as a standard Markov decision process when both *x* and *q* are included in the set of state variables. The optimal strategy is again chosen by maximizing *V* with respect to *A*, with the associated Bellman equation:

$$V\left(x_{t},q_{t}|A\right)=U\left(x_{t},q_{t},A\left(x_{t},q_{t}\right)\right)+\lambda \sum_{x_{t+1}}\sum_{k=1}^{K}q_{t}\left(k\right)P\left(x_{t+1}|x_{t},A\left(x_{t},q_{t}\right)\right)V\left(x_{t+1},q_{t+1}|A\right)$$ (7)

A key issue in determining the way optimal decisions are identified concerns the updating of the model state *q* in the decision process. In the adaptive management literature a distinction is made between active and passive adaptive management [22]. In active adaptive management the optimal policy is determined assuming that *q*_*t*_ is updated optimally using Bayes Theorem. We concentrate here on the passive form, in which the optimal policy is determined at each decision point under the assumption that the model state is fixed at current values (*q*_*t*+1_ = *q*_*t*_). Although the model state is assumed to be fixed in determining the policy, in practice it is updated at each time period, and the problem is resolved given the new model state. Thus the updating of the model state occurs outside the optimization algorithm, after a decision is implemented and system response *x*_*t*+1_ is recorded. At that time a new model state *q*_*t*+1_ is derived from *x*_*t*+1_, and another optimization is conducted based on the updated model state. With this sequence it is clear that at any particular time the choice of an action is influenced by both the current system state *x* and model state *q*. However, the choice is not influenced by the anticipated impacts of decisions on future model state (i.e., learning). In this sense, adaptive decision making is held to be passive. We note, however, that Eq (7) is for the active form and could be used if computational cost was not a concern. This is typically not true if there are more than a few alternative models because model state has to be carried along with system state in the optimization. Fortunately, we have found passive adaptive optimization to be a good approximation of the active form, often providing only slightly lower objective values [50, 51].

**Fig 1:**
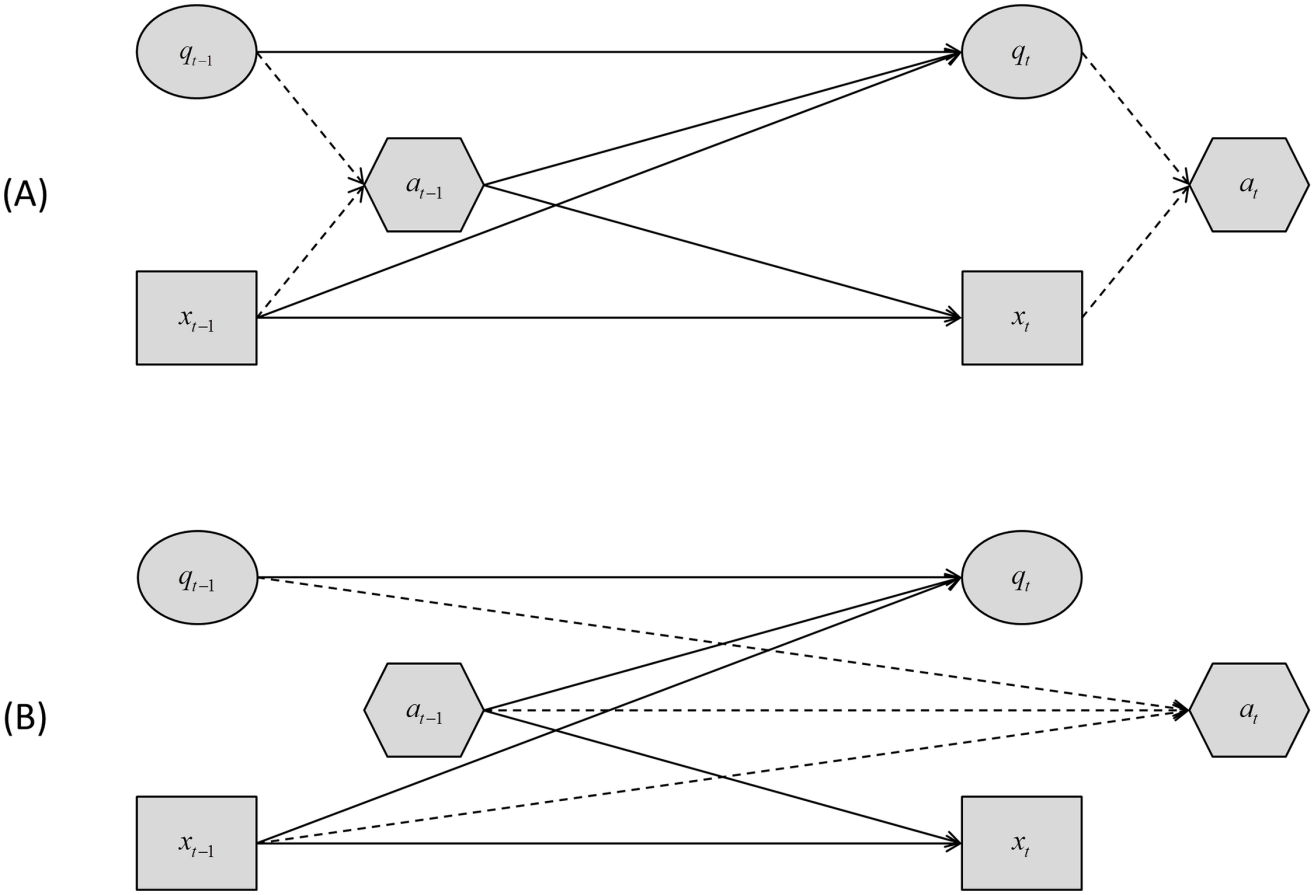
Markov decision process for decisions made immediately after (A) and before (B) the observation of system state. System state at time *t* is represented by *x*_*t*_, model state by *q*_*t*_, and actions by *a*_*t*_. The top panel (A) represents the post-survey decision and the bottom panel (B) represents the pre-survey decision. The solid arrows indicate the variables influencing the system and model states, while the dashed arrows indicate the variables influencing the action taken. Note that only the dashed arrows change between the two panels as the true system does not depend on the information known to the decision maker but only on the action the decision maker chooses.

**Fig 2:**
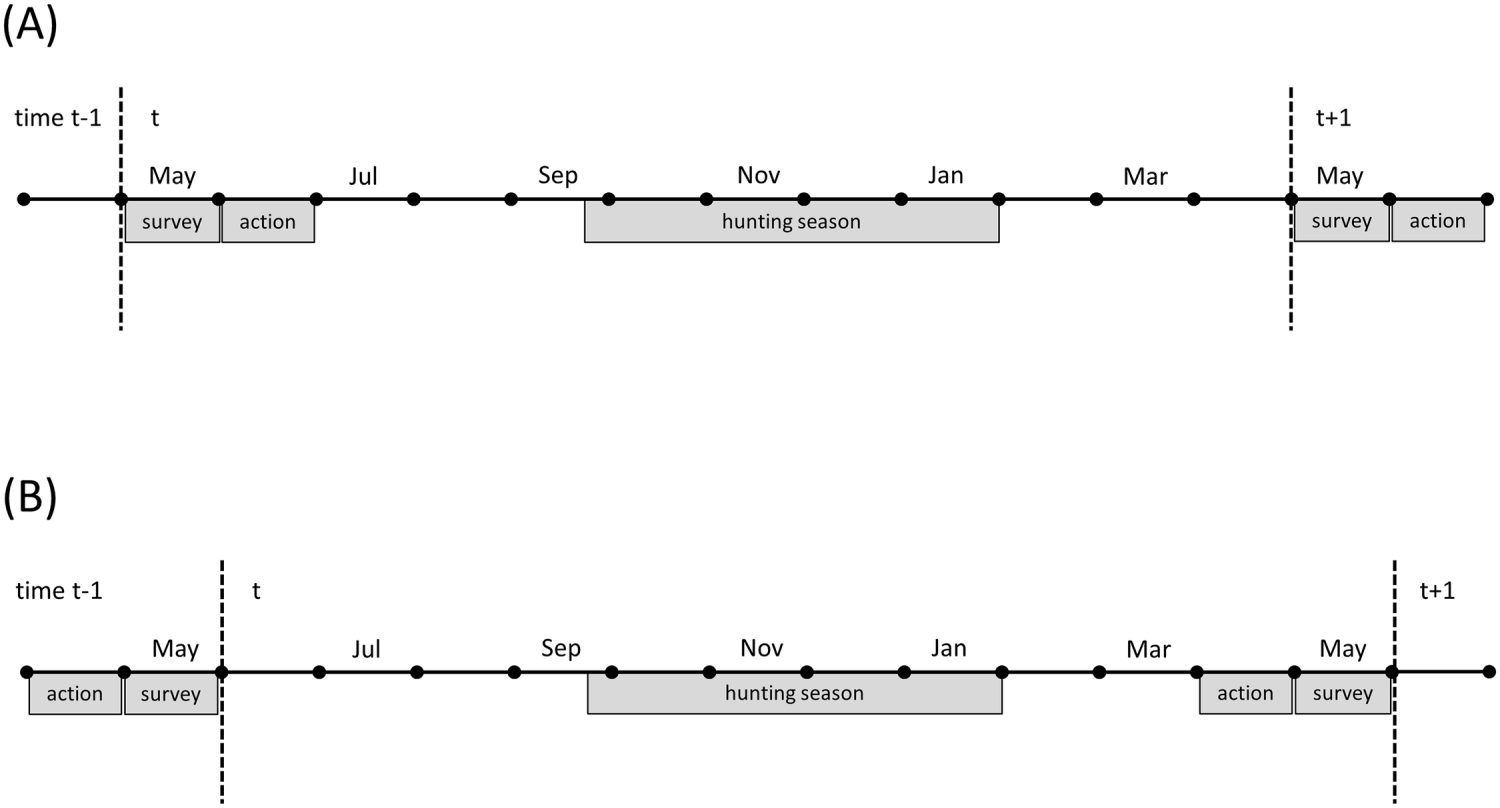
Timing of post-survey (A) and pre-survey (B) harvest management actions for waterfowl in the United States.

The above formulation is for the case where the decision and action occur immediately or soon after the response of the system to the previous decision has been observed via a monitoring program; we will refer to the policy resulting from this process as a post-survey policy (Fig 1). In the formulation of pre-survey policies, however, we must account for the fact that an action must be chosen before the effects of the last decision on system state are observed (Fig 2). The delay in observing system state until after the time when decisions are made has important implications. Consider decision making at a particular time *t*. The simplifying assumption that system state *x*_*t*_ is observed at the time the decision is made and the time *t* action taken allows the new model state *q*_*t*_ to be computed via Bayes’ theorem, so that an action can be determined with Eq (7). On the other hand, if recognition of system state *x*_*t*_ is unavailable at the time when an action is to be identified and taken, neither *x*_*t*_ nor the updated model state *q*_*t*_, which depends on *x*_*t*_, is available. The implication is that direct estimates of both state variables in Eq (7) are unavailable for the determination of an action. An approach under these circumstances is to base decision making on the most recent information that is available at time *t*, namely *x*_*t*−1_, *q*_*t*−1_, and *a*_*t*−1_. In particular, the model-specific utility *U*_*k*_(*x*_*t*_, *a*_*t*_) in Eq (7) can be replaced by the utility:

$$U_{k}(x_{t-1},a_{t-1,}a_{t})=\sum_{x_{t}}P_{k}(x_{t}|x_{t-1},a_{t-1})U_{k}(x_{t},a_{t}),$$ (8)

which in turn can be averaged over the models to give:

$$U(x_{t-1},q_{t-1},a_{t-1,}a_{t})=\sum_{k=1}^{K}q_{t-1}(k)U_{k}(x_{t-1},a_{t-1,}a_{t}).$$ (9)

This implies that the strategy *A* is now a function of the previous system and model states and the previous action: *a*_*t*_ = *A*(*x*_*t*−1_, *q*_*t*−1_, *a*_*t*−1_), such that the expected utility is:

$$U\left(x_{t-1},q_{t-1},a_{t-1},a_{t}\right)=\sum_{x_{t}}\sum_{k=1}^{K}q_{t-1}\left(k\right)P_{k}\left(x_{t}|x_{t-1},a_{t-1}\right)U_{k}\left(x_{t},a_{t}\right)$$ (10)

and the Bellman equation is:

$$\begin{array}{ll} V\left(x_{t-1},q_{t-1},a_{t-1}|A\right) & =U\left(x_{t-1},q_{t-1},a_{t-1},A\left(x_{t-1},q_{t-1},a_{t-1}\right)\right)+\\ & \lambda \sum_{x_{t}}\sum_{k}^{K}q_{t-1}\left(k\right)P_{k}\left(x_{t}|x_{t-1},a_{t-1}\right)V\left(x_{t},q_{t},A\left(x_{t-1},q_{t-1},a_{t-1}\right)|A\right). \end{array}$$ (11)

Note that an action is now conditioned on previous system and model states and also on the previous action, all of which are needed to determine the probability associated with the current system state, which is not known when the current decision is made and action taken. Thus, in practice, the current action is conditioned on an expectation of an objective value at the time the action must be chosen. Carrying the previous decision as a state variable preserves the Markov property of the decision process when the choice of an action must occur before the effects of the last decision are observed. Once again, this can be viewed as a traditional MDP in which the states now include the previous system state, model state, and action.

### Application to mallard harvest

The SEIS offered several alternatives concerning the timing of the regulatory process for setting waterfowl hunting seasons in the United States. The no-change alternative involved a process by which most proposals for hunting seasons are developed in response to monitoring information that becomes available in early summer, such as breeding population size, habitat conditions, and the previous season’s harvest. However, this leaves little flexibility in the timetable for setting hunting regulations prior to the opening of seasons in September. The preferred alternative of the SEIS is to advance this timetable by approximately three months, to allow more time for public input, provide earlier notification of the season’s regulations, and save time and money in administering the process (Fig 2). On the other hand, the regulatory decision must now be made in the absence of current-year monitoring information.

We here briefly describe the analytical framework used by the U.S. Fish and Wildlife Service to manage the harvests of mallards that breed in central Canada and the north-central United States ([33]). The data and analyses used by the U.S. Fish and Wildlife Service to develop this framework are provided as supporting information (S1 and S2 Files). The focus is on mallard abundance and the trajectory of population size through time. The state variable *x* consists of the population level and the number of ponds available for breeding and the action *a* is the regulatory decision about the level of allowable harvest.

Alternative models of population dynamics share a common form, which predicts changes in breeding-population size as a function of annual survival and reproductive rates [52]:

$$N_{t+1}=\gamma_{S}N_{t}\left\{mS_{t,AM}+\left(1-m\right)\left[S_{t,AF}+\gamma_{R}R_{t}\left(S_{t,JF}+S_{t,JM}\phi_{F}^{sum}/\phi_{M}^{sum}\right)\right]\right\}\cdot \text{exp}\left(\varepsilon^{N}\right)$$ (12)

where *N* is breeding population size, *m* is the proportion of males in the breeding population, *S*_*AM*_, *S*_*AF*_, *S*_*JF*_, and *S*_*JM*_ are annual survival rates of adult males, adult females, juvenile females, and juvenile males, respectively, *R* is reproductive rate, defined as the fall age ratio (young per adult) of females, $\phi_{F}^{sum}/\phi_{M}^{sum}$ is the ratio of female (*F*) to male (*M*) summer survival, γ denotes estimated bias-correction factors for survival (*S*) and reproduction (*R*), respectively, and *ε*^*N*^ is a normally distributed process error with mean 0 and variance 0.0184, and *t* is year. For optimization purposes we used five Gauss-Hermite quadrature nodes and weights to specify a discrete probability distribution for this process error [53].

Two alternative hypotheses for the relationship between annual survival and harvest rates are considered. For both models, survival in the absence of harvest is assumed to be the same for adults and juveniles of the same sex. In the model where harvest mortality is additive to natural mortality:

$$S_{t,sex,age}=s_{0,sex}^{A}\left(1-K_{t,sex,age}\right)$$ (13)

and in the model where the mortality process is compensatory:

$$S_{t,sex,age}=\left\{\begin{array}{ll} s_{0,sex}^{C} & \;if\;K_{t,sex,age}\leq 1-s_{0,sex}^{C}\\ 1-K_{t,sex,age} & if\;K_{t,sex,age}>1-s_{0,sex}^{C} \end{array}\right\}$$ (14)

where *s*_*0*_ = survival in the absence of harvest under the additive (*A*) or compensatory (*C*) model, and *K* is harvest rate adjusted for a crippling loss of 20% [54]. For the additive model, *s*_*0*_ = 0.7896 and 0.6886 for males and females, respectively. For the compensatory model, *s*_*0*_ = 0.6467 and 0.5965 for males and females, respectively. These estimates may seem counterintuitive because survival in the absence of harvest should be the same regardless of the model for annual survival under consideration. However, estimating a common *s*_*0*_ for both models leads to alternative models that do not fit available band-recovery data equally well. More importantly, it suggests that the greatest uncertainty about survival rates is when harvest rate is within the realm of experience. By allowing *s*_*0*_ to differ between additive and compensatory models, managers acknowledge that the greatest uncertainty about survival rate is its value in the absence of harvest.

Annual reproductive rates were estimated from the ratio of juvenile to adult females in the harvest, corrected using a constant rate of differential vulnerability derived from band-recovery data. Predictor variables are the number of ponds in May in Prairie Canada (*P*, in millions) and the size of the breeding population in the survey area. The best-fitting linear model was estimated with least-squares, and the 80% confidence ellipsoid for all model parameters was calculated [55]. Two points on this ellipsoid with the smallest and largest values for the effect of breeding-population size were used to generate a strongly density-dependent model:

$$R_{t}^{S}=\text{max}\left(0.0,1.1390+0.1376P_{t}-0.1131N_{t}\right)$$ (15)

and a weakly density-dependent model:

$$R_{t}^{W}=\text{max}\left(0.0,0.7166+0.1083P_{t}-0.0373N_{t}\right)$$ (16)

The two mortality and two reproductive models are combined into four alternative population models, which capture key uncertainties regarding the effects of harvest and environmental conditions on mallard abundance. The mortality hypotheses express different views about the effects of harvest on annual survivorship. The theoretical underpinning of the compensatory hypothesis is density-dependent mortality, in which mortality due to hunting is offset by declines in non-hunting mortality. The reproductive hypotheses represent alternative views regarding the degree to which per-capita reproductive rate declines with increases in mallard abundance and, thus, are also expressions of density-dependent population regulation. In 2015, model weights were 0.0011 for the compensatory survival / strong density-dependent reproduction model (ScRs), 0.3024 for the compensatory / weak model (ScRw), 0.0104 for the additive / strong model (SaRs), and 0.6861 for the additive / weak model (SaRw).

Finally, annual variation in pond numbers is modeled as a first-order autoregressive process:

$$P_{t+1}=\text{max}\left(0.0,2.2127+0.3420P_{t}+\varepsilon^{P}\right),$$ (17)

where ponds are in millions and *ε*^*P*^ is a normally distributed process error with mean 0 and variance 1.257. Again we used five quadrature nodes and weights to discretize the process error.

The United States Fish and Wildlife Service each year considers four regulatory alternatives, which encompass permissible season dates, season lengths, and daily bag limits. The expected harvest rates of mallards under each of the regulatory alternatives (closed, restrictive, moderate, and liberal) are updated annually using band-recovery information. Estimated harvest rates on adult male mallards in 2015 were 0.0088 (SD = 0.0020), 0.0552 (SD = 0.0129), 0.0977 (SD = 0.0215) and 0.1139 (SD = 0.0179) for closed, restrictive, moderate, and liberal hunting seasons, respectively. Normal distributions for these rates are assumed, and we again used five quadrature nodes and weights to discretize these harvest-rate distributions to account for a lack of perfect control over harvest. Predictions of harvest rates for the other age and sex cohorts are based on historic ratios of cohort-specific harvest rates to adult-male rates. These ratios were considered fixed at their long-term averages of 1.5407, 0.7191, and 1.1175 for young males, adult females, and young females, respectively.

The objective of mallard harvest management is to maximize the undiscounted, average annual harvest over an infinite time horizon, subject to a penalty if the expected population size falls below a target. Specifically,

$$\underset{\left(a_{t}|x_{t},q_{t}\right)}{\text{argmax}}U\left(x_{t},q_{t},a_{t}\right)=E\left[\alpha (N_{t+1})H_{t}|x_{t},q_{t},a_{t}\right]$$ (18)

and

$$\alpha (N)=\left\{\begin{array}{ll} 1.0 & \;if\;N\geq 8.5\\ N/8.5 & if\;N<8.5 \end{array}\right\}\;,$$ (19)

where total harvest *H* and population size *N* are in millions of mallards. Thus, the objective function proportionally devalues harvest actions that are expected to produce a population size less than 8.5 million in the subsequent year. In addition, there is a constraint in which closed seasons are not considered as long as the mallard population at the time of the decision satisfies *N*_*t*_ ≥ 4.75 million. In contrast to the post-survey decision problem, in the pre-survey decision problem the expectation in the utility is conditioned on *x*_*t*−1_, *q*_*t*−1_, *a*_*t*−1_ and *a*_*t*_. Furthermore, the closed-season constraint in the pre-survey decision problem must be imposed based on projected population size, *E*[*N*_*t*_|*x*_*t*−1_, *q*_*t*−1_, *a*_*t*−1_], because the current population size is not observed at the time of the decision. To avoid any confusion, we note that the objective function and closed-season constraint described here have only recently been adopted by the U.S. Fish and Wildlife Service and differ slightly from those it used for setting the 2016 hunting season (http://www.fws.gov/migratorybirds/pdf/management/AHM/SEIS&AHMReportFinal.pdf).

We used stochastic dynamic programming [56] and the set of software tools MDPSolve (https://sites.google.com/site/mdpsolve/) to compute optimal policies for the post- and pre-survey scenarios. MDPSolve was written in the Matlab programming language (http://www.mathworks.com/products/matlab/) and is freely available. In all cases, we specified an infinite time horizon, a discount factor *λ* = 1 (consistent with long-term sustainability), and value-function iteration and the average-reward criteria to calculate stationary, state-dependent policies [46, 56]. To solve for the optimal policy the state variables were discretized using increments of 0.125 over the interval 0.5 to 18 for population size and 0.5 to 8 for pond numbers (a total of 144 and 64 discrete values for these variables). The model state was discretized in increments of 0.1 (11 points for each model for a total of 286 model states).

We investigated differences between post- and pre-survey policies and expected performance for the two additive-harvest models (SaRs and SaRw) and for model weights in 2015. The two models with compensatory harvest mortality lead to the most liberal harvest regulation for all system states in both the post- and pre-survey scenarios. This is because all regulation-specific harvest rates are below the threshold of additivity mortality in these models. We used Monte Carlo simulations to derive expected performance characteristics of the passively adaptive policy under each of the four alternative models, assuming that each model in turn was the “correct” model. We ran 10 thousand replications of each simulation of 50 years. In each year of the simulation, model weights were updated based on a comparison of model predictions and the model-specific projection of population size. Then the appropriate action for the updated model state was imposed at the next decision. We calculated the mean annual population size and harvest, the annual probability of selecting each alternative action, and the mean model state (i.e., probabilities of each of the alternative models). The MDPsolve function “xpomdpsim” was use to perform simulations. This function simulates the discretized system model but uses the Bayesian updating rule to update the beliefs, with the action determined using the optimal action for the nearest discrete belief value. This prevents the simulated beliefs from prematurely reaching perfect certainty concerning which of the alternative models is correct.

## Results and Discussion

**Fig 3:**
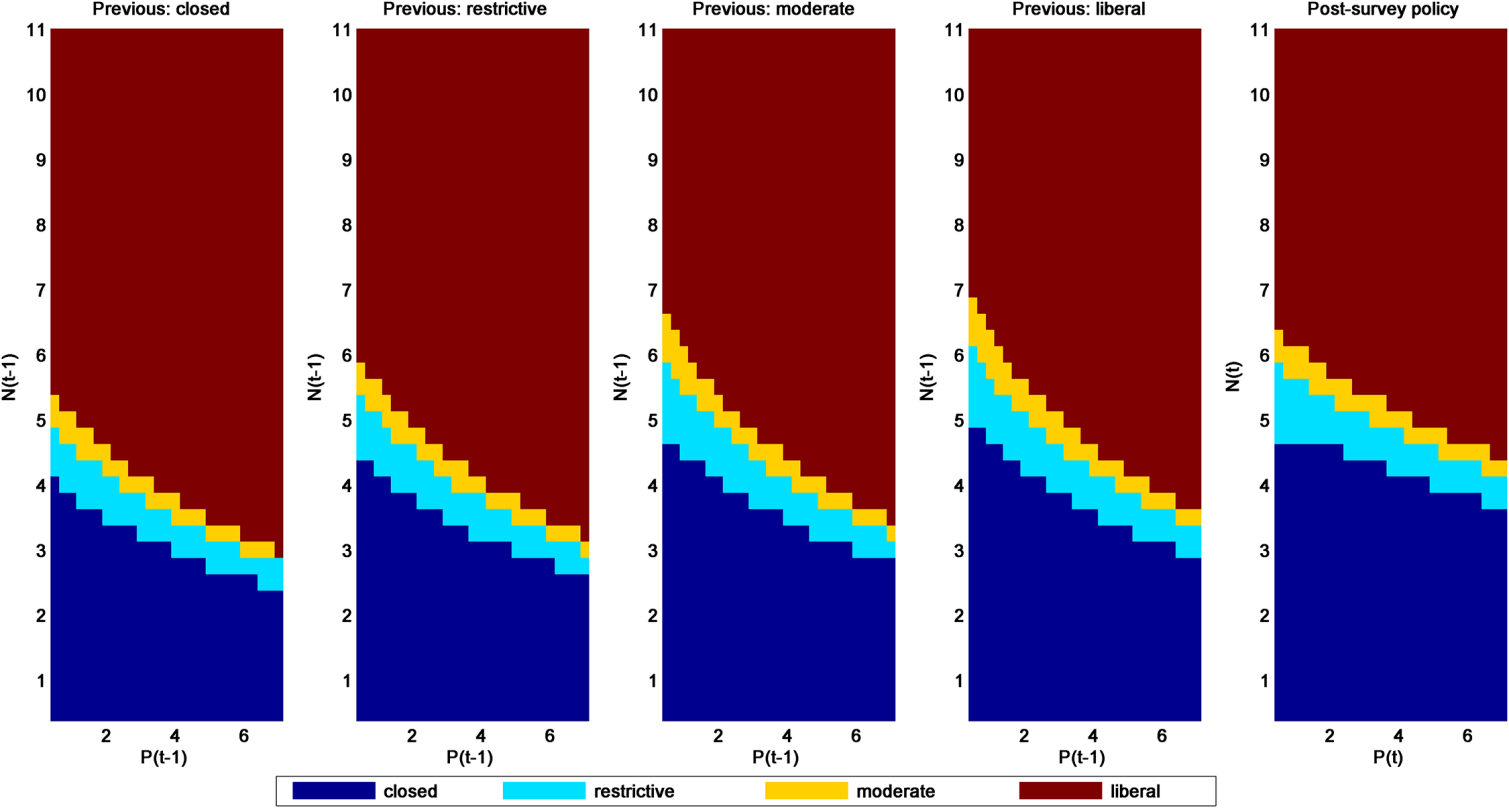
Pre- and post-survey harvest policies for the mallard model with additive hunting mortality and strong density-dependent reproduction (model SaRs).

**Fig 4:**
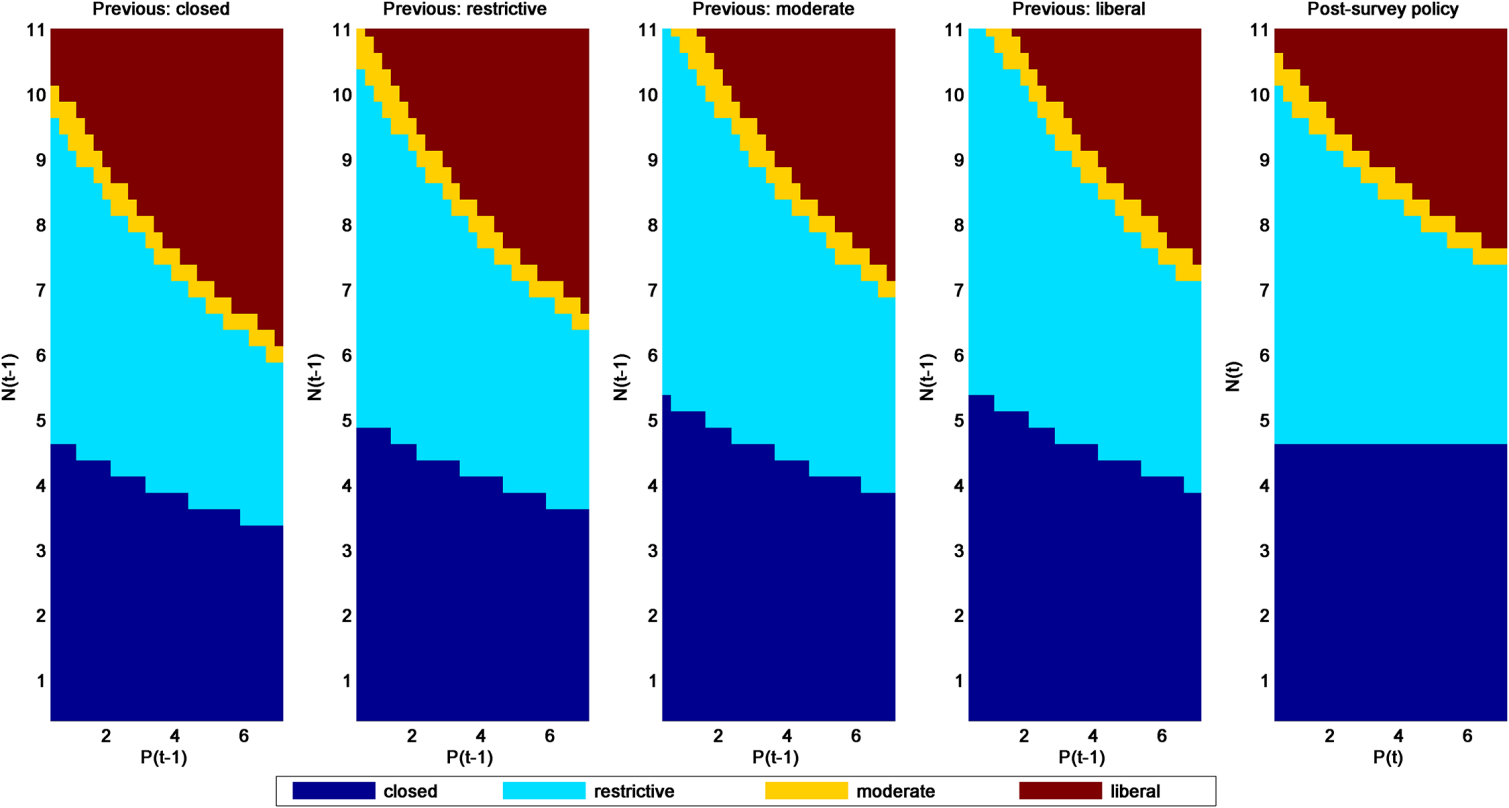
Pre- and post-survey harvest policies for the mallard model with additive hunting mortality and weak density-dependent reproduction (model SaRw).

Several patterns are noteworthy in the post- and pre-survey policies for the additive-mortality models (SaRs and SaRw in Figs 3 and 4, respectively). The most obvious is that the policies associated with the SaRs model are considerably more liberal than those for SaRw, regardless of whether one uses the post- or pre-survey policies. All else being equal, models with strong density dependence will lead to more liberal harvest policies than those with weak density dependence. Given uncertainty about the most appropriate model, these differences are a strong motivation for applying adaptive management to this population. Second, the pre-survey regulatory prescriptions generally become more liberal as the previous regulation becomes more conservative. This result occurs because a more restrictive season in the previous year would mean a relatively higher population in the following year when a new regulatory alternative must be chosen (at least under these additive-mortality models).

Another observation is that closed seasons are prescribed differently in the post- and pre-survey policies. For example in Fig 4, if the last observation of ponds was relatively low, then closed seasons can be prescribed even for (the last) observed population *N* ≥ 4.75 million. This is because low observed population and pond abundance in the previous year can result in an expectation of population size *E*[*N*] < 4.75 million at the time of the decision. Conversely, relatively high numbers of mallards and ponds observed in the previous year can result in an expectation *E*[*N*] ≥ 4.75 million and thus negate the prescription for a closed season.

A final pattern that is apparent in comparing post- and pre-survey policies is that the observed level of ponds tends to have more of an effect on regulatory prescriptions in the pre-survey policies, especially at the extremes. Pond numbers influence the mallard population through their effect on reproductive success. Hence, current pond numbers influence both the size of the current harvest and the level of next year’s population. When the current population level and pond numbers are unknown, the previous year’s pond numbers are predictive for the current population level and pond numbers. This additional source of influence on both current harvest and future population leads to a greater sensitivity of the optimal policy to the pond numbers.

**Fig 5:**
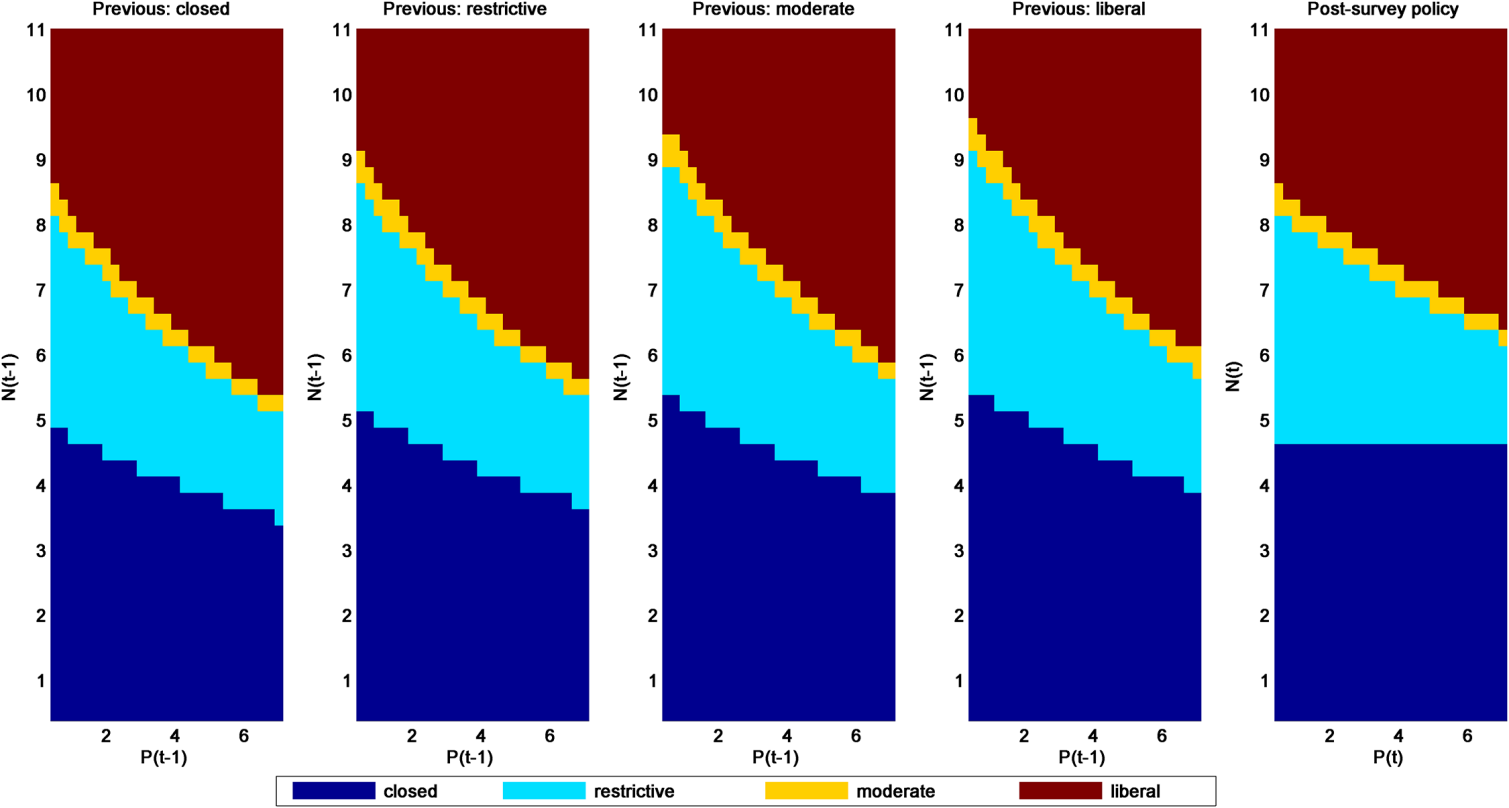
Pre- and post-survey policies for the four alternative mallard models using passive adaptive optimization and model weights from 2015 (0.0104, 0.6861, 0.0011 and 0.3024 for the SaRs, SaRw, ScRs and ScRw models, respectively).

The passive adaptive policy (Fig 5) can be seen as intermediate among the four model-specific policies, but is heavily influenced by the SaRw model, which had a relatively high weight (0.6861) in 2015. Otherwise, patterns are very similar to those in the SaRw and SaRs models.

**Fig 6:**
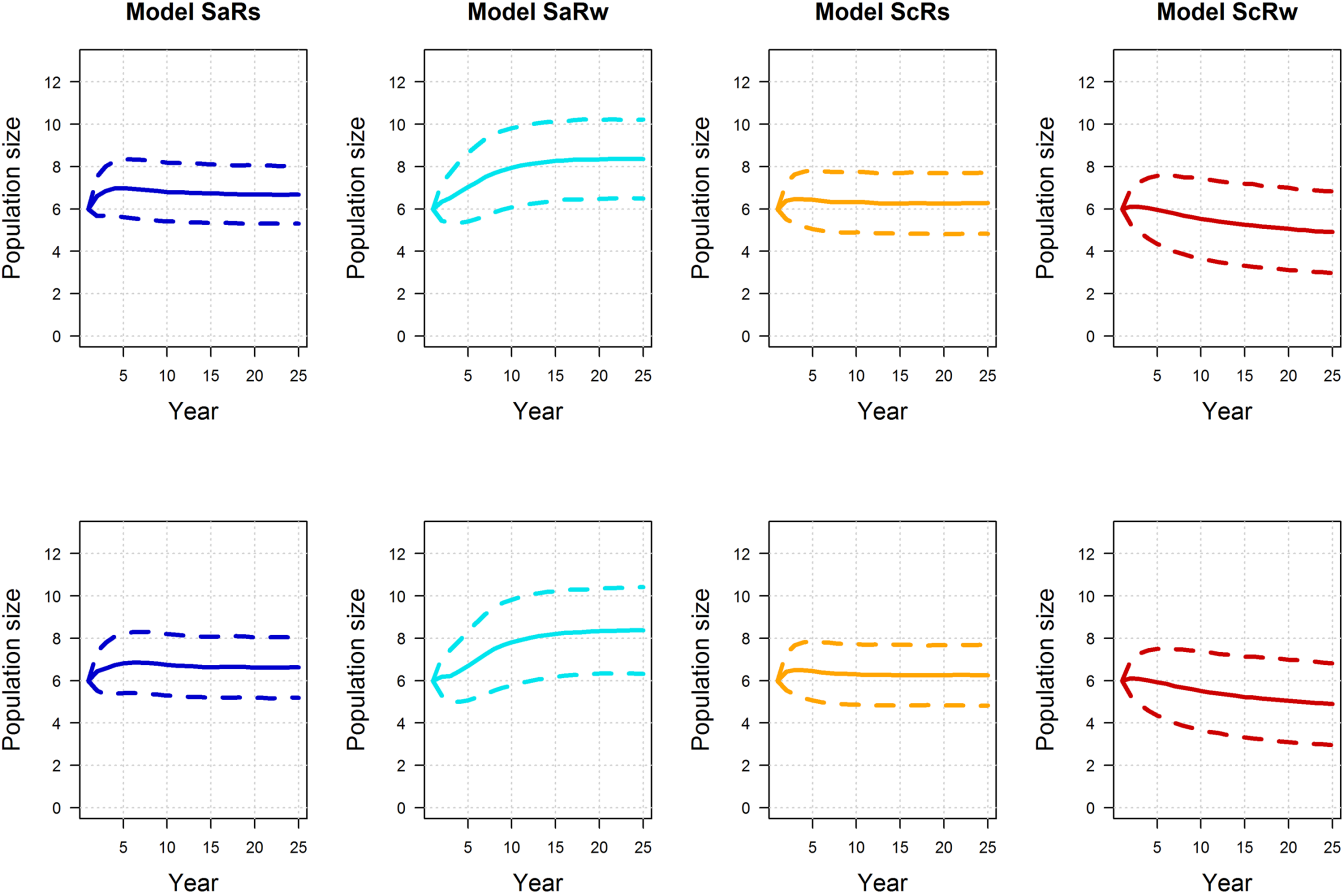
Simulated, model-specific expectations of population size for post (1^st^ row) and pre-survey (2^nd^ row) passively adaptive policies, assuming the “correctness” of four alternative models of mallard population dynamics (SaRs = additive mortality and strong density-dependent reproduction; SaRw = additive mortality and weak density-dependent reproduction; ScRs = compensatory survival and strong density-dependent reproduction model; and ScRw = compensatory survival and weak density-dependent reproduction). The dotted lines represent the mean +/- one standard deviation.

**Fig 7:**
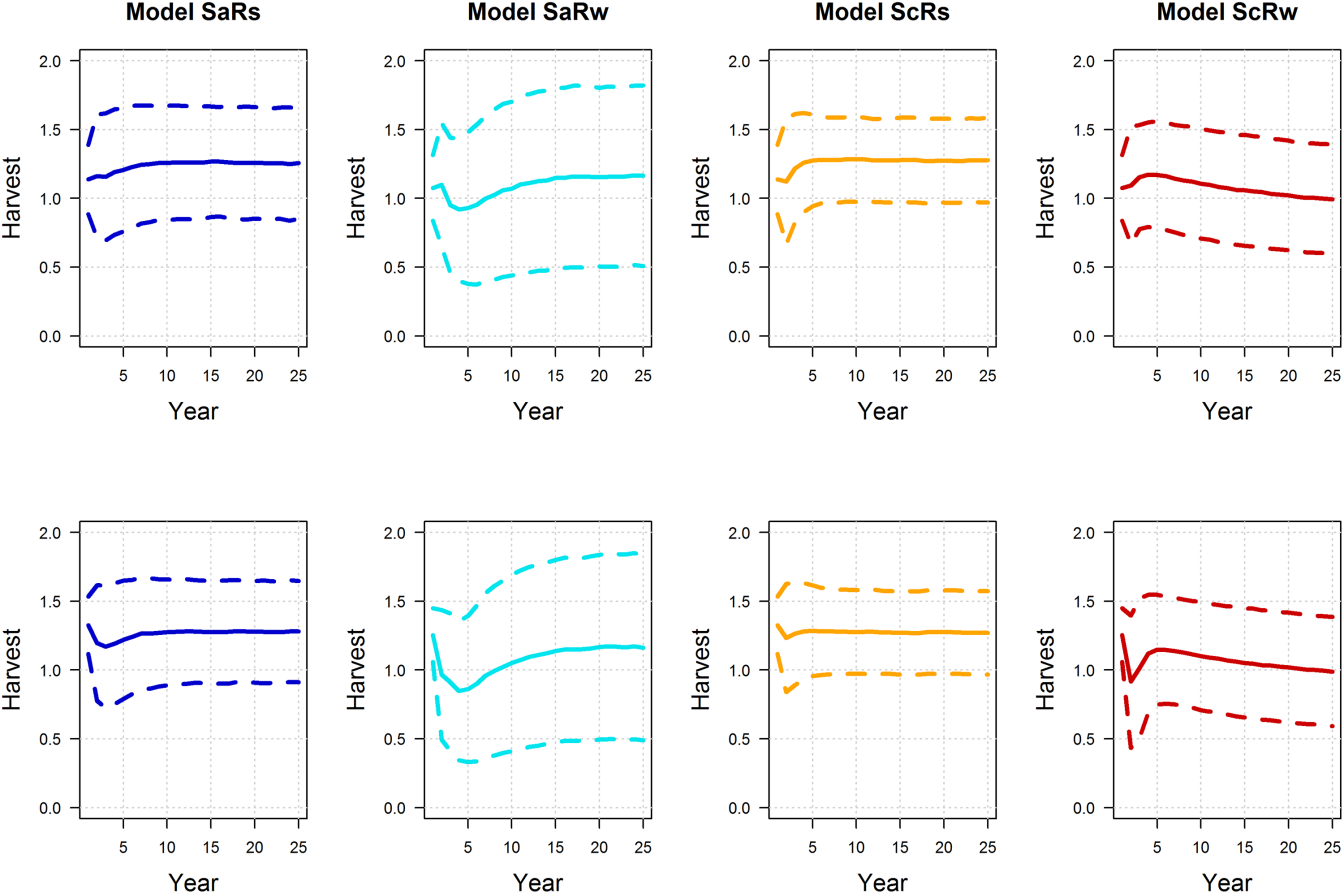
Simulated, model-specific expectations of harvest under for post (1^st^ row) and pre-survey (2^nd^ row) passively adaptive policies, assuming the “correctness” of four alternative models of mallard population dynamics (SaRs = additive mortality and strong density-dependent reproduction; SaRw = additive mortality and weak density-dependent reproduction; ScRs = compensatory survival and strong density-dependent reproduction model; and ScRw = compensatory survival and weak density-dependent reproduction). The dotted lines represent the mean +/- one standard deviation.

**Fig 8:**
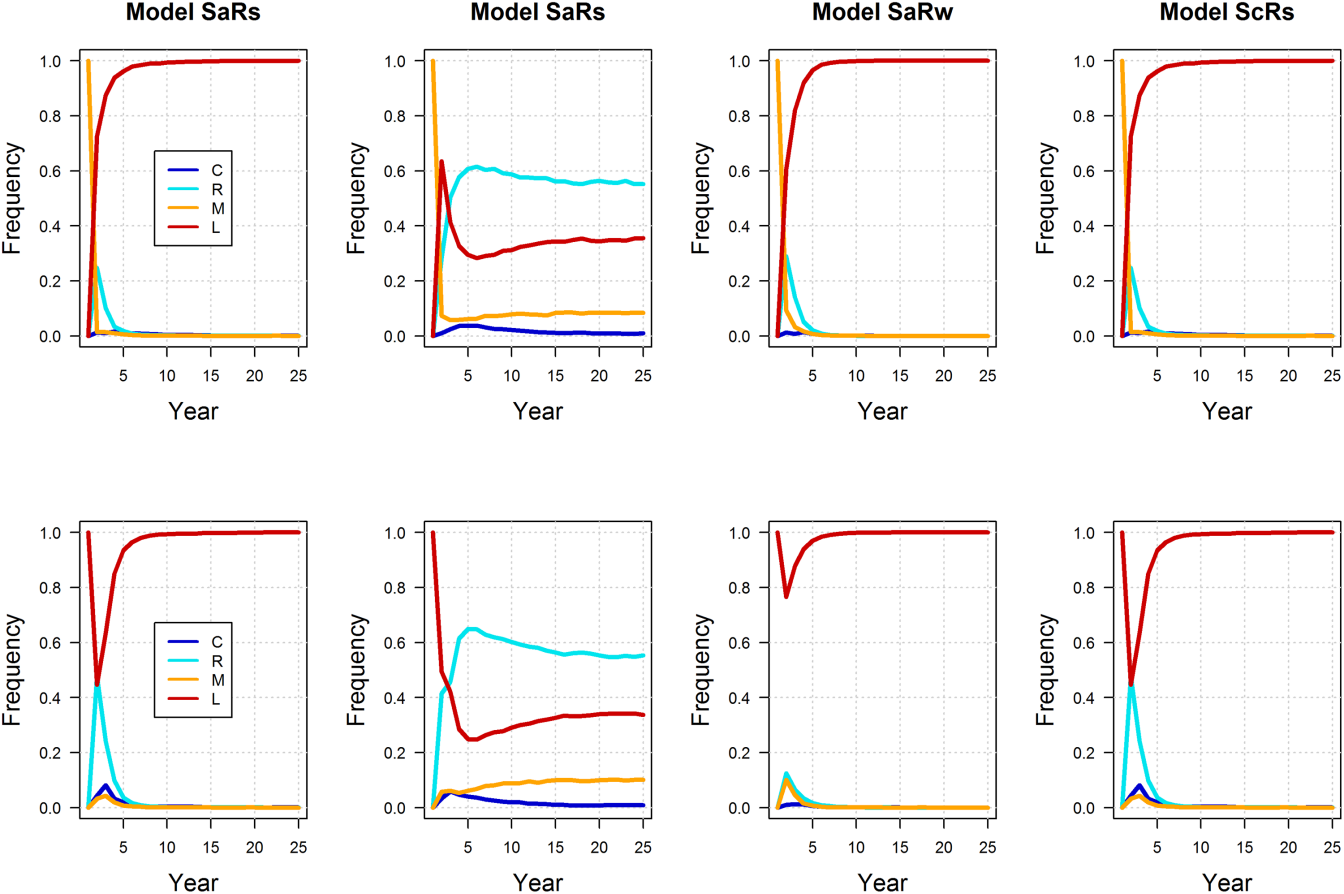
Simulated, model-specific expectations of regulatory actions (C = closed, R = restrictive, M = moderate, and L = liberal) for post (1^st^ row) and pre-survey (2^nd^ row) passively adaptive policies, assuming the “correctness” of four alternative models of mallard population dynamics (SaRs = additive mortality and strong density-dependent reproduction; SaRw = additive mortality and weak density-dependent reproduction; ScRs = compensatory survival and strong density-dependent reproduction model; and ScRw = compensatory survival and weak density-dependent reproduction).

**Fig 9:**
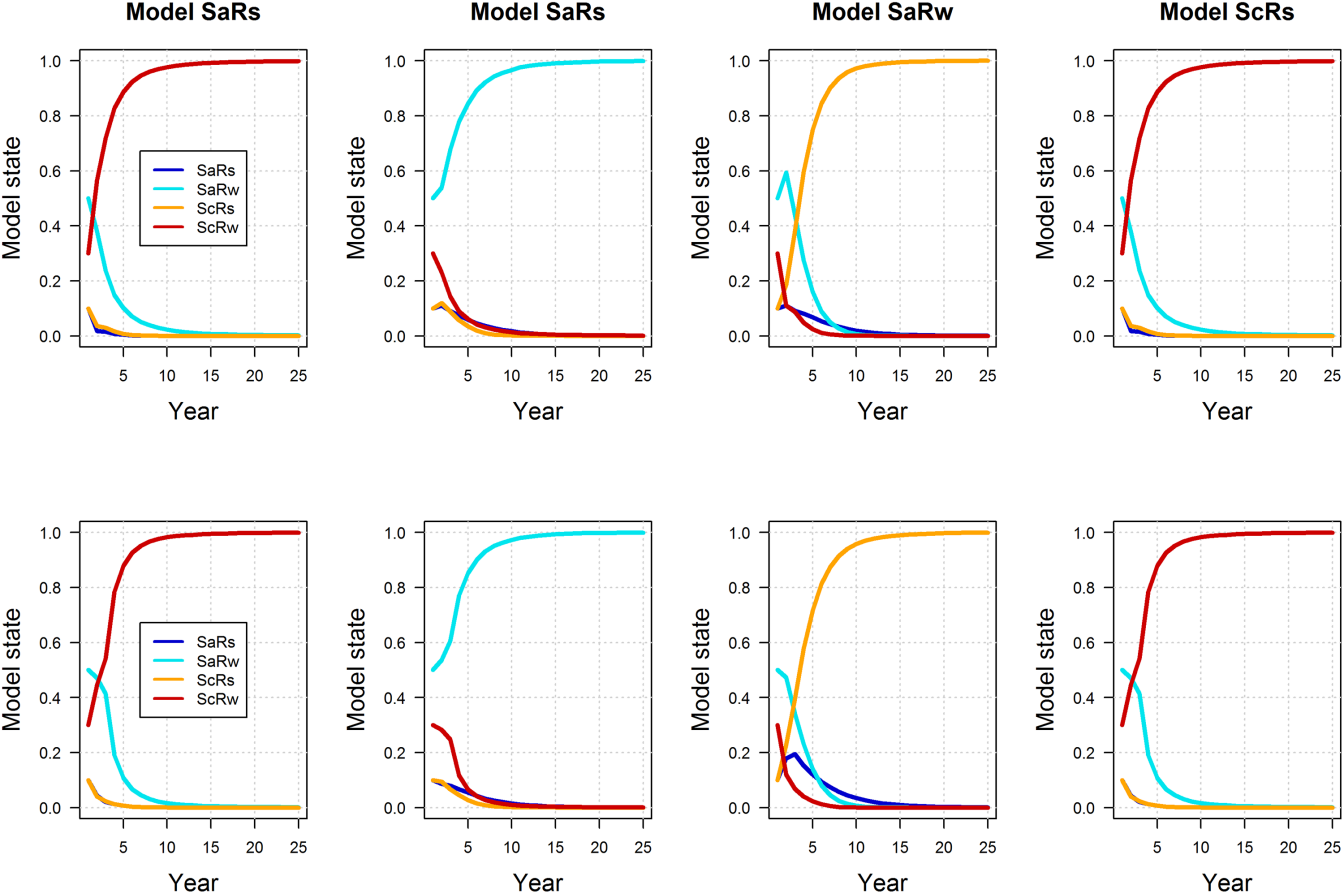
Simulated, model-specific weights for post (1^st^ row) and pre-survey (2^nd^ row) passively adaptive policies, assuming the “correctness” of four alternative models of mallard population dynamics (SaRs = additive mortality and strong density-dependent reproduction; SaRw = additive mortality and weak density-dependent reproduction; ScRs = compensatory survival and strong density-dependent reproduction model; and ScRw = compensatory survival and weak density-dependent reproduction).

Interestingly, the post and pre-survey policies lead to small differences in expected performance. Model-specific expectations of mean population size were similar between post and pre-survey policies, with marginally more variation under the pre-survey policy, but only for the models with additive hunting mortality (recall that under the compensatory models, harvest has no effect on population size) (Fig 6; note that the small difference in population variation is not readily apparent in the figure). Model-specific expectations of long-term mean harvest are also very similar between post and pre-survey policies, but there is some indication of less harvest under the pre-survey policy in the early years of the timeframe (Fig 7). Accordingly, it’s in the early part of the timeframe that we see differences between the post and pre-survey policies in the regulatory actions chosen (Fig 8). The simulations were initialized with the first period having a population of 6 and pond numbers of 4 for the post-survey case and lagged population, pond numbers and action of 6, 4 and liberal for the pre-survey. The differences in then initial conditions led to the early differences between the two cases. The simulations were also initialized so the model state was equal to [0.10.50.10.3]. These values are close to current model weights but are chosen to be exactly at one of the values used to discretize the model state. From this initial point the passive adaptive policy was able to learn which model was “correct” within about 20 years, regardless of whether the post or pre-survey policy was used (Fig 9). Again, the difference in performance between policies occurred in the early years of the timeframe.

## Conclusions

Our pre-survey decision-making framework is applicable to any number of situations where for administrative or logistical reasons decisions must be based on monitoring information that is not current. For mallards, we found rather intuitive patterns in the optimal harvest policies when shifting from a post- to a pre-survey decision-making framework. However, we were surprised that there were not larger differences in the expected performance of the two frameworks. In the case of mallards, we speculate that the small differences are due to the (modeled) resilience of the population, the lack of strong transient population behavior, and possibly the relatively narrow range of harvest rates resulting from the current regulatory alternatives. And, in our case, decisions are made with a time lag measured only in months. But in some situations, monitoring may be done less frequently than the time step of decision making (a simple extension of our framework could account for this). In these cases we should expect an increasing erosion of performance as the status of the system at the time of decision and action becomes more uncertain.

When using a finite set of models in a decision-making context an important assumption is that the model set contains the “correct” model (i.e., a model that closely approximates the dynamics of the system). The simulation results concerning the revisions of model weights (Fig 9) suggest that the “correct” model should be known with near certainty within about 20 years. Given that the adaptive management program has been conducted since the mid-1990s, the “correct” model should now be known. The fact that one model has not emerged as dominant suggests that the model set may not be rich enough and that none of the models provides a close approximation of system dynamics [57]. Managers are currently developing a richer set of alternative models for this system. There are also novel solution methods that can account for many models so that the probability is high that the model set contains the “correct” model [58], as well as methods that can account for the fact that the “correct” model might change over time [59].

It is worth comparing our approach with the Partially Observable Markov Decision Process (POMDP) approach that has been used to address problems in which state variables are imperfectly observed [60]. The POMDP approach maintains a belief distribution for the unobserved state variables and updates it based on the state transition probabilities and any other available relevant information. In the current case, however, all relevant information concerning the current (unobserved) state is contained in the values of the previous state and action variables and the system model(s). It is therefore not necessary to maintain a belief distribution. It should be noted, however, that a POMDP framework could be used to address both observational and structural uncertainty in an adaptive management framework, especially if an active adaptive management strategy is desired [58, 59, 61–65] The framework used here obviates the need for a POMDP approach because the conditional probability distribution for the current year is assumed to be known given the previous states and actions. This would no longer be true, however, if additional information, such as harvest levels of mallards or information about the weather influencing pond numbers, was used to condition probabilities about the current states [62]. A POMDP would also be needed if there was an explicit accounting for sampling variation in population and pond surveys.

Finally, while we believe our approach for coping with lagged information about system states has utility, there are some important caveats. Augmenting the state space with the decision made in the previous time step introduces additional complexity and computational cost, particularly in the case of partial controllability (i.e., those cases where there is stochasticity in control variables, such as the relationship between mallard hunting regulations and harvest rate) [14]. More critically, the interpretation and communication of patterns in a pre-survey policy can be difficult because the number of state variables has to increase by one to accommodate the previous decision made. However, this difficulty is true of any policy in which the number of state variables ≥ 3 because of the difficulty in depicting the policies graphically. We suggest that the use of classification trees [66] might be useful in such cases to graph an approximate, but more easily interpreted, policy.

## Supporting Information

S1 File A revised protocol for the adaptive harvest management of mid-continent mallards. (PDF)

S2 File Data used to develop a revised protocol for the adaptive harvest management of mid-continent mallards. (XLS)
