# Supplementary material for: State-Dependent Resource Harvesting with Lagged Information about System States
Source: PLoS One. 2016 Jun 17;11(6):e0157373. doi: 10.1371/journal.pone.0157373 (PMC4912162; doi:10.1371/journal.pone.0157373)
Supplement: S1 File — (PDF) [file pone.0157373.s001.pdf]

A Revised Protocol
for the
Adaptive Harvest Management of Mid-continent Mallards

June 17, 2002

Michael C. Runge
U.S. Geological Survey, Patuxent Wildlife Research Center

Fred A. Johnson
U.S. Fish and Wildlife Service, Division of Migratory Bird Management

James A. Dubovsky
U.S. Fish and Wildlife Service, Region 6

William L. Kendall
U.S. Geological Survey, Patuxent Wildlife Research Center

Jeff Lawrence
Minnesota Department of Natural Resources

Jim Gammonley
Colorado Division of Wildlife

Abstract

The methods and models used for the adaptive harvest management (AHM) of mid-continent mallards have been reviewed and revised. This revision was motivated by recognition of two important concerns about the current methods: bias at the "balance equation" level arising from unknown bias in underlying monitoring data; and an underestimate of the variance of the predictions from the models, due to the omission of several components of the variance. In the revised set of models: (1) the balance equation bias is corrected by including an empirical adjustment to the survival and reproductive rates; (2) the survival sub-models are re-parameterized to better reflect uncertainty about the additive and compensatory hypotheses; (3) the recruitment sub-models are re-estimated; and (4) the prediction error includes all variance components, and is inflated to reflect uncertainty in the total variance of predicted population size. These revisions to the AHM protocol suggest that the optimal strategy, using the current regulatory alternatives, will call for liberal regulations less often in the Mississippi, Central, and Pacific Flyways than it has in the past, with a corresponding increase in the frequency of more conservative regulations. The revisions also have resulted in different conclusions about the best predictive models for mid-continent mallards—the model weights suggest much less evidence for the hypotheses of additive hunting mortality and strongly density-dependent reproductive rates compared to results using the old protocol.

BACKGROUND

The population models upon which harvest regulations for midcontinent mallards are based have been in place since 1995. The basic structure of the models, alternative hypotheses of population dynamics, and support for each hypothesis (i.e., model "weights") are subject to continuous review by parties both internal and external to the AHM process. For the last two years, the AHM Working Group (AHMWG) has been focusing on two especially important concerns about the existing models for mid-continent mallards, and is making certain revisions this year.

Apparent bias in reproductive or survival rates.—The current population models for mid-continent mallards share a common structure referred to as the balance equation. The balance equation is essentially an accounting tool, which predicts population size in a given year based on population size (N), reproduction (R), and survival (S) from the previous year. In theory, N, R, and S from a given year should perfectly predict N the next year. In fact, they do not (Fig. 1). Predicted population sizes are higher on average than those observed in the population surveys. The source and cause of the bias in estimates of survival and reproductive rates are unknown, but data-collection programs are being carefully scrutinized.

Updating model weights.—The purpose of annually updating model weights is to eventually identify the model providing the most accurate predictions over time, based on a comparison of the observed population size with those predicted under each alternative model. Model weights are highly influential in determining optimal harvest strategies because they determine the degree to which a single set of biological hypotheses (i.e., a particular model) dominates the optimization. The AHM Working Group has identified a shortcoming in the current procedure for updating model weights due to the omission of certain random errors common to all predictive models. The inclusion of these prediction errors in the updating procedure will minimize the chances of major shifts in model weights in any single year, and help ensure that model weights accurately support the model(s) with the best predictive ability.

The purpose of this report is to describe the AHM Working Group's efforts to address these modeling issues. Final decisions regarding modification of the model set for mid-continent mallards will be made after the USFWS has discussed resulting management implications with the Flyway Councils, States, and the general public.

DATA SETS

Reproductive Rates

To estimate fall age ratios of mid-continent mallards (defined as those banded in areas encompassed by the traditional survey area of the Waterfowl Breeding Ground Population and Habitat Survey [or May aerial survey, MAS], strata 1-18, 20-50, and 75-77), we first wanted to ensure that the various sources of data we used described approximately the same population of birds. We used Munro and Kimball (1982) to identify the states in which ∃80% of the harvest was derived from birds in the MAS (banding reference areas 1-7, 12-13 [Anderson and Henny 1972]). We obtained the harvest data from 1961-1995 for those states from the Division of

Migratory Bird Management (unpub. data), and divided the sex-specific annual harvests of young by those of adults to estimate the harvest age ratios.

We then obtained banding and recovery files for mallards banded during 1961-95 from the U.S. Geological Survey's Bird Banding Laboratory (BBL). We used only data from normal, wild mallards that were banded during July-September, and that were shot or found dead during the hunting season. For birds banded within the MAS and the states of Michigan, Minnesota, and Wisconsin, we calculated annual direct recovery rates (Anderson and Henny 1972) and annual harvest rates (i.e., direct recovery rates divided by band-reporting rates) for each cohort, to use in estimating fall age ratios (i.e., harvest age ratios divided by the differential vulnerability of young relative to adults). To estimate harvest rates, we used estimates of band-reporting rates reported by Nichols et al. (1995). However, because Nichols et al. (1995) did not have sufficient numbers of banded birds to estimate reporting rates for all age and sex classes, we imputed values for the missing cohorts (C.T. Moore, unpub. data).

We calculated fall age ratios (young/adult) for males and females separately using the following 3 methods, ranging from very fine-grained (method 1) to coarse-grained (method 3) analyses:

Method 1 ("Fall M1").—We used the harvest rates calculated above (using geographic variation in band-reporting rates) to estimate cohort-specific harvest rates for each year and banding reference area. We then calculated, from the MAS data, the proportion of the spring mallard population in each of the banding reference areas for each year. Next, we multiplied the year-, cohort-, and reference-area specific harvest rates by the population proportions, and summed these products to estimate annual, population-weighted harvest rates for each cohort. Finally, we divided the harvest rates of young by those for adults to obtain annual vulnerability estimates. To estimate annual fall age ratios, we divided the annual harvest age ratios by the annual vulnerability estimates.

Method 2 ("Fall M2").—We calculated annual direct recovery rates for each cohort and divided the recovery rate of young by that for adults to estimate annual vulnerability estimates for each reference area. As in the first method, we calculated the proportion of the spring mallard population in each of the banding reference areas for each year. We then multiplied the year- and reference-area-specific estimates of vulnerability by the corresponding population proportion. Finally, we summed these products within each year to obtain annual population-weighted estimates of vulnerability. The annual harvest age ratios were divided by the annual vulnerability estimates to estimate annual fall age ratios.

Method 3 ("Fall M3").—We calculated annual direct recovery rates pooled over reference areas for each cohort and divided the recovery rate of young by that for adults to estimate annual vulnerability estimates. The annual harvest age ratios were divided by the annual vulnerability estimates to estimate annual fall age ratios.

We also considered using fixed, rather than annual, vulnerability adjustments. To calculate these fixed adjustments, we averaged the annual vulnerability estimates for 1961-1995 for each of the three methods described above. These fixed adjustments were then divided into the annual harvest age-ratios to produce three additional fall age-ratio series for each sex.

Survival Rates

Age- and sex-specific survival rates for each year, 1961-95, were computed under model H1 from Brownie et al. (1985). We included reference areas 2-6, 12, and the part of areas 13 and 14 that matched with the MAS area. We could not include reference areas 1 and 7 due to sparse data. We computed annual survival estimates for the entire mid-continent in each of two ways. First, we pooled data from all references listed above to produce one survival estimate for each year. Second, we conducted separate analyses for each reference area, and then averaged the survival rates, weighting each estimate by population size for that reference area based on the MAS. Here we did not include area 14 because abundance estimates were not available for a sufficient number of years for MN, MI, and WI.

We obtained band recovery data from the USGS Bird Banding Laboratory. We used only data from normal, wild mallards banded July-September and shot or found dead during the hunting season. Solicited and unsolicited band recoveries were included.

Kill Rates

Age- and sex-specific kill rates for each year, 1961-86, were computed from band recoveries, based on a survival model that partitions annual survival rate into survival during the hunting season and survival during all other times of the year combined (Johnson et al. 1993). We conducted separate analyses for each banding reference area and then averaged results across reference areas, weighting by population size from the MAS. We included reference areas 2-6, 12, and the part of area 13 that is included in the MAS. We ignored reference areas 1 and 7 due to sparse data, and area 14 because abundance estimates were not available for a sufficient number of years for MN, MI, and WI.

We used only normal wild mallards banded July-September and shot or found dead during the hunting season. To convert recovery rates to kill rates we assumed crippling loss of 20% (Anderson and Burnham 1976). To account for reporting rates we incorporated geographical variation in estimated reporting rate based on Nichols et al. (1995). We incorporated the reward and control band data from that 1988-91 study directly into our analysis. Reward bands and solicited bands were assigned a reporting rate of 1.0 throughout the analysis.

Other Data

Breeding Population Size.—We used the May breeding population size (BPOP) estimates from the traditional mid-continent mallard strata (breeding reference areas 1-7, 12, and 13, Anderson and Henny 1972). We did not include the “Lake States” (Minnesota, Wisconsin, and Michigan) in the BPOP totals for analysis because those state surveys began later (1968, 1973, and 1992, respectively) than the surveys for the traditional areas. For model-building purposes, we used BPOPs from 1961-1996. For model updating, we used data from 1996-2001.

Ponds.—We considered two sets of pond data: the traditional estimate of May ponds (Canadian ponds only), and estimates of total ponds (including U.S. ponds). The Canadian pond data is

available from 1961-present, and the U.S. (and thus, total) pond data is available from 1974-present.

DEVELOPING AND CORRECTING THE BALANCE EQUATION MODEL

The Balance Equation, Anniversary Dates, and Sex Ratio

The annual survival rates and the fall age-ratio can be combined in a model that accounts for changes in the population size (Fig. 2). Because the midpoint of the banding is August, the survival rate estimates encompass the period August_t to August_{t+1}. Thus, the population model most naturally has an anniversary date of August. A fraction, m_P, of the pre-harvest adult population is male (total size of the adult male pre-harvest population is P_{AM}), the remainder, $1 - m_P$, is female (total size, P_{AF}). The age-ratios, R_t, estimate the number of immature females per adult female in the pre-harvest population. If we assume an equal sex ratio at hatching and equal summer survival for male and female juveniles, then the number of juveniles in the pre-harvest population is given by $2R_tP_{AF}$. We assume that half of these juveniles in the fall are female, and if they survive, become adult females in the following year. The annual survival rates for all of the age/sex classes ($S_{t,\text{ age/sex}}$) are estimated from the banding data, and can be applied to the numbers in each class to predict the number of adult males and females in the August population in the following year.

In order to compare the predictions from this population model to observed changes in the population size as measured by the MAS, we needed to change from an August-to-August population model to a May-to-May population model (Fig. 3). Two assumptions were needed to make this transition: (a) adult summer survival (from May to August) is constant over time, but may differ by sex; and (b) the summer survival of first-year birds is the same as that of adults of the same sex. The BPOP is divided into males (N_{AM}) and females (N_{AF}) with a male fraction of m (different from the pre-harvest male fraction, m_P). Since the age-ratios are measured in the fall flight, a female must survive the summer (with survival rate ϕ_F^{sum}) in order to successfully reproduce. The survival rates of juveniles cover only a portion of the annual cycle (from August to May), and so must have the summer survival portion of them removed (hence the division by ϕ_F^{sum} and ϕ_M^{sum} in Fig. 3). Aggregating these elements, the predicted number of females in the next year is

$$\begin{aligned} N_{t+1,AF} &= BPOP_t(1-m)\left[S_{t,AF} + 2\phi_F^{sum}R_t \cdot 0.5S_{t,JF} \big/ \phi_F^{sum}\right] \\ &= BPOP_t(1-m)\left[S_{t,AF} + R_tS_{t,JF}\right] \end{aligned}, \qquad (1)$$

the predicted number of males is

$$\begin{aligned} N_{t+1,AM} &= BPOP_t\left[mS_{t,AM} + (1-m)2\phi_F^{sum}R_t \cdot 0.5S_{t,JM} \big/ \phi_M^{sum}\right] \\ &= BPOP_t\left[mS_{t,AM} + (1-m)R_tS_{t,JM}\left(\phi_F^{sum} \big/ \phi_M^{sum}\right)\right] \end{aligned}, \qquad (2)$$

and the full "balance equation" that predicts the breeding population size in the next year is

$$BPOP_{t+1} = BPOP_t \left\{ mS_{t,AM} + (1-m)\left[S_{t,AF} + R_t \left(S_{t,JF} + S_{t,JM}\, \phi_F^{sum} / \phi_M^{sum} \right) \right] \right\}. \quad (3)$$

Thus, to predict the BPOP in the next year, in addition to the survival rates, age-ratio, and current BPOP, we needed estimates of the male fraction (*m*) and the ratio of female to male summer survival ($\phi_F^{sum}/\phi_M^{sum}$, note that we only needed to know the ratio, not the sex-specific values). In this model, we assumed that both of these quantities were fixed and known, a necessary assumption because there are not operational programs to monitor either quantity. We estimated the inverse of the summer survival ratio ($\psi = \phi_M^{sum}/\phi_F^{sum}$) by using the results of the survival sub-model estimation (see "Survival Sub-models", below). We assumed that differences in survival in the absence of harvest were due to differences in summer survival between sexes (i.e., that the post-harvest to May survival was the same for both sexes). For the additive survival model,

$$\psi = \frac{\phi_M^{sum}}{\phi_F^{sum}} = \frac{\phi_M^{sum}\phi^{win}}{\phi_F^{sum}\phi^{win}} = \frac{s_{0,M}}{s_{0,F}} = \frac{0.7896}{0.6886} = 1.147. \quad (4)$$

For the compensatory survival model, $\psi = 1.084$. For the balance equation calculations, we averaged these two values, and used $\psi = 1.115$.

To estimate *m*, the male fraction in the breeding population, we expressed the balance equation in matrix form

$$\begin{bmatrix} N_{t+1,AM} \\ N_{t+1,AF} \end{bmatrix} = \begin{bmatrix} S_{AM} & RS_{JM}/\psi \\ 0 & S_{AF} + RS_{JF} \end{bmatrix} \begin{bmatrix} N_{t,AM} \\ N_{t,AF} \end{bmatrix}, \quad (5)$$

substituted the mean values for the survival rates and age-ratios, and calculated the right eigenvector of the transition matrix. The right eigenvector gives the stable sex structure of a population growing according to equation 5 with constant survival and reproductive rates. The mean values for the survival and reproductive rates were from the data sets selected below (see "Choosing the Data Sets", $S_{AM} = 0.6932$, $S_{AF} = 0.6196$, $S_{JM} = 0.6203$, $S_{JF} = 0.5932$, and $R = 0.8256$). With a ratio of male to female summer survival rates of $\psi = 1.115$, the estimated May male fraction obtained from the eigenvector was 0.5246. The corresponding August male fraction (m_P), calculated in the same manner (but dropping ψ from the equation) was 0.5517.

Choosing the Data Sets

As noted above ("Data Sets"), we had multiple survival and age-ratio datasets to consider. In the process of revising the mid-continent mallard models, we had one primary goal: to increase the predictive ability of the models. This goal applied to selection of the datasets, as well as to all subsequent decisions about the models. To evaluate the predictive ability of the datasets, we ran each possible combination of survival and age-ratio datasets through an August-to-August balance equation (equation 3 without the summer survival ratio, and with *m* set to 0.55) to predict a *relative change* in population size ($\lambda_t = P_{t+1}/P_t$). We then compared these predicted

changes in population size with the observed changes in population size from the May Aerial Survey ($BPOP_{t+1}/BPOP_t$), calculating a correlation coefficient over the 35-year time series (1961-1995). While the anniversary dates of the predicted and observed changes do not match, we reasoned that we did not need to be concerned with the time scale adjustments here because those adjustments were all constant across years and would not greatly change the correlations.

We considered two annual survival datasets (*weighted* by reference area, and *pooled* across reference areas). We considered 12 different age-ratio datasets, differing by sex (female vs. male age-ratios), type of vulnerability adjustment (annual vs. fixed), and method of calculating vulnerabilities (M1, M2, or M3). The results for 5 of these summarize the results for the rest (Table 1). For calculating the correlation between predicted and observed changes in population size, a fixed vulnerability adjustment (no matter what the value of the vulnerability) gives the same results as using the raw harvest age-ratio. The results for only one of the male age-ratio datasets are shown, the rest follow the same pattern as the female age-ratio datasets.

The correlations between the predicted and observed population size changes ranged from 0.38 to 0.67 (Table 1). In all cases, the weighted survival-rate dataset outperformed the pooled survival rate dataset, female age-ratios outperformed the corresponding male age-ratios, and age-ratios using vulnerability adjustments based on direct recovery rates (M2 and M3) outperformed those using vulnerability adjustments based on estimated harvest rates (M1). Of considerable interest, age-ratios that included a fixed adjustment for vulnerability (represented in Table 1 by the harvest age-ratios) performed *better* than age-ratios that included an annual adjustment for vulnerability. This suggests that the reductions in bias that come from including annual vulnerability adjustments do not compensate for the increase in variance of vulnerability estimates.

Thus, the combination of datasets that resulted in the highest correlation between predicted and observed changes in population size contained the weighted survival rates and the fixed-vulnerability female age-ratios. But, there were *three* fixed-vulnerability female age-ratio datasets (differing in whether the vulnerability was based on M1, M2, or M3), all of which produced nearly the same correlation since they differed only by a multiplicative constant. Of the age-ratio datasets that used *annual* vulnerability adjustments, the dataset that used pooled direct recovery rates to estimate vulnerability (“Fall M3”) produced the highest correlation between predicted and observed population changes. This suggested to us that the vulnerability adjustments calculated with this method were the most accurate. Therefore, we calculated a mean vulnerability adjustment from the annual vulnerability adjustments produced by this method (1961-1995), and applied that constant vulnerability adjustment (1.751 young female:adult female) to the harvest age-ratios. The resulting set of fall age-ratios became the recruitment dataset we used in all subsequent analyses.

Estimating Bias in the Balance Equation

When the chosen survival and recruitment datasets were used in a May-to-May balance equation (equation 3, with m = 0.5246 and ψ = 1.115), the predicted population sizes ($BPOP_{t+1}$) were greater on average than the corresponding observed population sizes (Fig. 1). The average over-prediction was 10.8%.

We considered the following three hypotheses for the bias: (1) bias in the annual survival rates only, with all four age/sex classes having the same proportional bias; (2) bias in the age-ratios only; and (3) bias equally divided between survival rates and age-ratios. We expressed these hypotheses as modified balance equations. The survival-bias-only hypothesis suggests the following balance equation:

$$BPOP_{t+1} = \gamma_S BPOP_t \left\{ mS_{t,AM} + (1-m)\left[S_{t,AF} + R_t \left(S_{t,JF} + S_{t,JM} \, \phi_F^{sum} / \phi_M^{sum} \right) \right] \right\}. \quad (6)$$

The correction factor, γ_S, is the same for each survival rate and can be factored to the front of the equation. The recruitment-bias-only hypothesis suggests the following correction:

$$BPOP_{t+1} = BPOP_t \left\{ mS_{t,AM} + (1-m)\left[S_{t,AF} + \gamma_R R_t \left(S_{t,JF} + S_{t,JM} \, \phi_F^{sum} / \phi_M^{sum} \right) \right] \right\}. \quad (7)$$

Here, the correction factor, γ_R, adjusts only the age-ratio, not the entire equation. The hybrid hypothesis puts two correction factors into the equation, in the same positions as indicated in equations 6 and 7.

We used a least squares approach to estimate the correction factors. Using the annual (1961-1995) values for the age-ratios and four survival rates, the fixed constants *m* and ψ, and the observed May populations sizes ($BPOP_t$), we derived the values for the correction factors that minimized the sum of squared differences between the observed and predicted May population sizes ($BPOP_{t+1}$, 1962-1996). For the survival-bias-only hypothesis, the correction factor is γ_S = 0.8965 (SSE = 21.01×10^{12}). For the recruitment-bias-only hypothesis, the correction factor is γ_R = 0.7396 (SSE = 18.68×10^{12}). Note that, based on the observed data from 1961-1995, the recruitment-bias-only hypothesis is a better predictor than the survival-bias-only hypothesis, as evidenced by the smaller SSE.

To estimate simultaneous correction factors for a hybrid-bias hypothesis, we chose initial values for γ_S and γ_R that were midway between the single-source-bias values and 1. Then, conditioning on one of these initial values, we calculated the value for the other that minimized the sum of squared differences, as above. Conditional on γ_S = 0.9483, the SSE is minimized with γ_R = 0.8543. Conditional on γ_R = 0.8698, the SSE is minimized with γ_S = 0.9476. We took the midpoint between these two pairs of estimates. Thus, for the hybrid-bias hypothesis, we used γ_S = 0.9479 and γ_R = 0.8620. Use of these correction factors in the balance equation removes the obvious bias (compare Fig. 4 to Fig. 1).

We argue below (under "Optimal Equilibrium Points") that there is not a practical difference in performance between the survival-bias-only and recruitment-bias-only hypotheses. Thus, we have proposed retaining only the hybrid-bias hypothesis for developing the optimal policies and updating model weights. Hypotheses about the cause of the bias will continue to be examined outside of the formal adaptive management structure, both by examination of balance equation predictions and consideration of external studies. As such information becomes available, we anticipate that it will be incorporated into the mallard models.

DEVELOPING THE SUB-MODELS

Survival Sub-models

There were two reasons to rebuild the survival sub-models, rather than just retaining the ones used previously. First, because we were concerned about introducing additional sources of bias, we wanted the annual survival rates used in the balance equation analysis to be the response variable for development of the survival sub-models. Second, we wanted to reconsider the nature of our uncertainty about the survival rates (specifically, uncertainty about the survival rate in the absence of harvest).

In the development of the previous set of survival models (Johnson et al. 1997), band-recovery data were used in an ultra-structural model (Smith and Reynolds 1992) to estimate non-hunting-season survival, assuming a crippling loss of 0.2 (Anderson and Burnham 1976) and a band-reporting rate of 0.32 (Nichols et al. 1991). The estimated degree of additivity, β, was 0.93 (G.W. Smith, pers. comm.), indicating that the data showed substantially more support for the additive model than the compensatory model. The non-hunting-season survival calculated in this manner was then used as the basis for developing *both* the additive and compensatory models. That is, both models were assumed to have the same survival in the absence of harvest.

We believe there are two ways we can improve the set of survival sub-models. First, the previous additive and compensatory models did not fit the data equally well. Since the non-hunting-season survival was estimated assuming a largely additive model, yet applied to both models, the additive model fit the data well, but the compensatory model did not. Second, as an expression of uncertainty about the effects of hunting on survival, the previous strategy for developing the two survival sub-models suggests high certainty about the survival rate when the harvest rate is zero (because both models predict the same rate), but low certainty when the survival rate is in the realm of experience (because that's where the two models differ the most). We think the nature of the uncertainty is exactly the opposite. We have the most confidence about the annual survival rates when the harvest rate is in the realm of experience. We have the most uncertainty about annual survival rates when the harvest rate is zero, since we've never observed that situation.

To address these concerns, we took the following approach to developing the survival sub-models. For each reference area, we estimated the annual survival rates and kill rates for each age and sex (see "Data Sets" above). We assumed that survival in the absence of harvest was the same for adults and juveniles of the same sex (thus, we assumed that all the differences in annual survival rates for adults and juveniles was due to differences in kill rates). We considered two models for the relationship between annual survival rates and kill rates: the additive model

$$S_{t,sex,age} = s^{A}_{0,sex}\left(1 - K_{t,sex,age}\right), \quad (8)$$

and the compensatory model

$$S_{t,sex,age} = \begin{cases} s^{C}_{0,sex} & if\ K_{t,sex,age} \leq 1 - s^{C}_{0,sex} \\ 1 - K_{t,sex,age} & if\ K_{t,sex,age} > 1 - s^{C}_{0,sex} \end{cases} . \tag{9}$$

Independently for each model and each sex, we estimated the s_0 parameter by finding the value that minimized the sum of squared differences between the observed and predicted annual survival rates. For example, for males in reference area 2, the estimated non-hunting-season survival rates were 0.73 and 0.59 for the additive and compensatory models, respectively (Fig. 5B). If a common s_0 is assumed for the two models, and is chosen to minimize the sum of squared differences under both models simultaneously, the estimated non-hunting-season survival is 0.65 (Fig. 5A). However, note that estimating a common s_0 produces two models, neither of which fit the data very well—the additive model consistently under-predicts the annual survival rate, and the compensatory model consistently over-predicts the annuals survival rate. Instead, estimating the non-hunting-season survival rate independently (Fig. 5B) gives two alternative models that fit the data equally well.

To combine the estimates of non-hunting-season survival across reference areas (2-6, 12, 13), we weighted by the May population sizes (BPOPs). These weighted averages determined the two survival submodels used in subsequent steps. The additive survival submodel followed equation 8, with $s^{A}_{0,M}$ = 0.7896 and $s^{A}_{0,F}$ = 0.6886. The compensatory submodel followed equation 9, with $s^{C}_{0,M}$ = 0.6467 and $s^{C}_{0,F}$ = 0.5965.

These results seem counterintuitive because the true survival in the absence of harvest should be the same regardless of the model for the effect of harvest. But, we do not know the true survival rate in the absence of harvest. We have better knowledge of the annual survival rate under moderate to liberal harvest rates. Thus, we actually expect the survival in the absence of harvest to be higher under the additive model, because just as increasing kill rates reduces annual survival under the additive model, *decreasing* kill rates should *raise* annual survival. In contrast, under the compensatory model, reducing the kill rate below (1 – s_0) should have no effect on the annual survival rate. The important change here is that the point of reference should be our range of experience, namely annual survival rates under moderate to liberal kill rates, *not* annual survival rates in the absence of harvest. Uncertainty about the nature of the effect of harvest on survival implies uncertainty about the survival rate in the absence of harvest.

For several years, the AHM working group has been pursuing more mechanistic alternatives for the survival models (Johnson et al. 1993), such as alternatives that include density-dependence. Such investigations are not within the scope of this current set of revisions, but are underway.

Recruitment Sub-models

The primary reason for rebuilding the recruitment sub-models was to use the same set of age-ratios that were used in the balance equation analysis. We also reconsidered how to express the alternative hypotheses about the effect of breeding population size on recruitment.

The response variable for the recruitment analysis was female age-ratio, corrected using a constant vulnerability term (the same data set chosen for the balance equation analysis). The

predictor variables were May Canadian ponds and the breeding population size (BPOP). We fit a linear model to the data for 1961-1995 (Table 2), but did not reconsider the model selection procedures described in Johnson et al. (1997). Regression diagnostics did not indicate any violations of the assumptions of linear regression.

To generate alternative hypotheses, we calculated the 80% confidence ellipsoid for all the parameters (Draper and Smith 1981), using

$$(\beta-\mathbf{b})'\mathbf{X}'\mathbf{X}(\beta-\mathbf{b}) \le ps^2 F_{p,\nu,0.2} \quad (10)$$

where β is a point in the parameter space, **b** is the vector of least squares parameter estimates, **X** is the design matrix, p is the number of parameters, ν is the error degrees of freedom, and s^2 is the mean squared error. We chose the two points on this ellipsoid with the largest and smallest values for the BPOP slope to generate the weak and strong density-dependent models, respectively. The weak density-dependent model was

$$R_t = 0.7166 + 0.1083 Ponds_t - 0.0373 BPOP_t \quad (11)$$

and the strong density-dependent model was

$$R_t = 1.1390 + 0.1376 Ponds_t - 0.1131 BPOP_t\,. \quad (12)$$

Predicted values for these two models, at the mean value of 3.36 million ponds, are shown in Fig. 6.

The previous recruitment models (Johnson et al. 1997) were formed in a slightly different manner—the least squares estimate for the parameters was used as the weak density-dependent model, and the minimum on the 95% confidence ellipsoid was used as the strong density-dependent model. We felt that this created an unequal pair of models, since the two models were not equally supported by the data. Instead, we chose two models that were on the same confidence ellipsoid. We chose the 80% confidence ellipsoid because we felt it was the largest ellipsoid that produced predictions that were biologically reasonable.

We considered the use of “Total Ponds” (Canadian and U.S. May ponds) as a predictor instead of just Canadian May ponds. While Total Ponds explains more of the variance in recruitment, the time series is shorter (1974 forward), and this made the confidence ellipsoid substantially larger, made it difficult to estimate the autoregressive model for ponds (see “Ponds Sub-model” below), and increased the overall variance of the predictions in the model (see “Estimating Prediction Variance” below). Work is underway to consider a more comprehensive revision of the recruitment sub-models, including consideration of total ponds as well as other variables, and other functional forms. We expect development of new recruitment models within the next two years.

Ponds Sub-model

We modeled the annual variation in the Canadian May ponds (1961-1995) as a first-order autoregressive process. The autoregressive parameter was marginally significant ($P = 0.045$). The estimated model was

$$Ponds_{t+1} = 2.2127 + 0.3420 Ponds_t + \varepsilon_t \tag{13}$$

where ε_t is normally distributed with mean 0 and variance MSE = 1.2567 (ponds are in millions). The mean value for Canadian ponds in this model is 3.363 million.

This model improves upon the previous model for pond dynamics (Johnson et al. 1997) by (1) using a formal autoregressive-moving-average (ARMA) model to estimate the parameters, rather than an *ad hoc* linear regression approach, and (2) properly accounting for the full variance in annual changes in ponds (the previous model included variance due to fluctuations in rainfall, but omitted the mean squared error of the regression).

Differential Vulnerability to Harvest

The survival sub-models predict annual survival rates in four age-sex classes from kill rates of those same four classes. We use differential vulnerabilities relative to adult males to predict the kill rates of the other three age-sex classes. In the next section ("Harvest Rates"), we develop a model for predicting adult male kill rate as a function of the regulations package.

In order to maintain consistency, when developing estimates of differential vulnerability we used the same sex-, age-, year-, and reference area-specific kill rates that were used to develop the survival sub-models above. We calculated sex-, age-, year-, and reference area-specific differential vulnerabilities by dividing the appropriate kill rate by the corresponding kill rate for adult males. For each sex/age class and year, we weighted these differential vulnerabilities across reference areas (2-6, 12, 13) by BPOP. We then averaged these annual values (1961-1986) to arrive at the final sex- and age-specific vulnerabilities. These values were $d_{JM} = 1.5407$, $d_{AF} = 0.7191$, and $d_{JF} = 1.1175$.

Harvest Rates

The final sub-model predicts kill rates from the current regulatory alternatives. We relied on an existing assessment for regulation-specific predictions of the mean harvest rate (U.S. Fish and Wildlife Service, Adaptive Harvest Management: 2001 Duck Hunting Season, U.S. Dept. Interior, Washington, D.C., 47 pp.; available online at http://migratorybirds.fws.gov/mgmt/ahm/ahm-intro.htm). We assumed regulation-specific harvest rates were distributed normally, and that standard deviations were 20% of the mean (Johnson et al. 1997). We also used standard Bayesian statistical techniques to update the predicted harvest rates under the liberal alternative, based on observed harvest rates during the recent liberal seasons (Johnson, F. A., J. A. Royle, and M. C. Runge. 2002. Framework-date extensions and the adaptive management of mallard harvests, U.S. Fish and Wildlife Service, Washington, D.C.,10 pp.; available online at http://migratorybirds.fws.gov/mgmt/ahm/ahm-intro.htm). This updating, which was based on

harvest rates observed during the 1998-2001 hunting seasons, resulted in a decrease in the mean harvest rate under the liberal alternative from 0.1305 to 0.1210, and a decrease in the standard deviation from 20% to 18% of the mean. Harvest rates of cohorts other than adult males were predicted using the constant rates of differential vulnerability provided above. We also used a constant crippling loss rate of 0.2 to translate harvest rates to kill rates.

Lake States

The model development to this point has not included the Lake States (Wisconsin, Minnesota, and Michigan) in the measurements of the breeding population size, primarily because full data sets are not available from those areas to allow appropriate analysis. Lake States mallards have been included in the mid-continent mallard AHM models since 1997 by assuming that population dynamics for mallards in the Lake States are similar to those in the traditional mid-continent region. We recommend continuing these methods, that is, scaling up the predictions to reflect the proportionate increase in the population size due to the inclusion of the Lake States. Issues relevant to this adjustment include: (1) the recruitment sub-models use the breeding population size from the traditional area only, because these data were used to develop the sub-models; and (2) for the purposes of model weight updating (see "Model Weight Updating" below) we only use the population sizes from the traditional survey area.

From 1992 through 2001, when BPOP data were available for all Lake States, the proportion of the *total* mid-continent BPOP (reference areas 1-7, 12, 13, *and* 14) that was in the Lake States (reference area 14) had a mean of 0.1083 and a standard deviation of 0.0212. We assumed a normal distribution with these parameter values to make the conversion between traditional and total BPOPs.

ESTIMATING PREDICTION VARIANCE

Using the balance equation and sub-models provided above, predictions of breeding population size in year $t + 1$ depend only on specification of a regulatory alternative, an estimate of Canadian ponds in year t, and an estimate of population size in year t. For the period in which comparisons were possible, we were interested in how well these predictions corresponded with observed population sizes. In making these comparisons, we were primarily concerned with how well the bias-corrected balance equation and the reproductive and survival sub-models performed. Therefore, we relied on estimates of adult male kill rates (1961-1986) rather than regulatory alternatives as inputs (effectively separating the partial controllability component from the rest of the model).

We assumed that the errors in the predictions from the overall model were multiplicative and lognormally distributed, that is

$$\ln BPOP_{t+1}^{obs} \sim N\left(\ln BPOP_{t+1}^{pred}, \sigma^2\right). \qquad (14)$$

We estimated σ^2 by comparing the observed BPOPs with those predicted by the overall model,

$$\hat{\sigma}^2 = \left(\frac{1}{n-1}\right)\sum_t \left(\ln BPOP_t^{obs} - \ln BPOP_t^{pred}\right)^2 . \quad (15)$$

We were concerned about obtaining a variance estimate that was too small, either by chance, or because our time series was too short. Therefore, we calculated an 80% confidence interval for the variance

$$\frac{(n-1)\hat{\sigma}^2}{\chi^2_{n-1,1-\alpha/2}} < \sigma^2 < \frac{(n-1)\hat{\sigma}^2}{\chi^2_{n-1,\alpha/2}} \quad (16)$$

and used the upper value as our prediction variance (equivalent to multiplying $\hat{\sigma}^2$ by 1.518, since n = 26) . We obtained four estimates of the prediction variance, depending on whether we assumed survival was additive or compensatory, and whether recruitment was strongly or weakly density-dependent. We took the square root of each variance and averaged over the four sets of assumptions. The final estimate for the total prediction error was $\hat{\sigma}$ = 0.1558. This is equivalent to a coefficient of variation of about 17%.

The previous estimate of variance was developed by simulating the contributions of several variance components, including the estimates of sampling error from the May aerial surveys, but it did not include error from the balance equation or the recruitment or survival sub-models. Our new method for estimating variance improves upon the old method in three ways—the new variance estimate: (1) has a formal distribution (equation 14), rather than an empirical distribution, and so avoids the inherent problem of estimating tail probabilities (P.B. Conn and W.L. Kendall, pers. comm.); (2) includes all possible sources of variance, because it is estimated holistically rather than piecemeal; and (3) is suitably inflated to guard against underestimation. While this larger variance will appear to slow learning, it more accurately reflects the uncertainty in our predictions, and so we expect it will reduce the dramatic shifts in model likelihoods we observed when updating model weights previously.

PROPERTIES OF THE FINAL MODEL SET

Based on the analyses described above, we specified a revised set of models for mid-continent mallards that includes four alternatives:

ScRs: compensatory survival, and strongly density-dependent reproduction;
ScRw: compensatory survival, and weakly density-dependent reproduction;
SaRs: additive survival, and strongly density-dependent reproduction;
SaRw: additive survival, and weakly density-dependent reproduction.

All four of these alternative models share the same balance equation, which has bias corrections equally apportioned between survival rates and age-ratios. The four models also use the same prediction variance.

Optimal Equilibrium Points

We used the methods of Runge and Johnson (2002) to calculate the optimal equilibrium points for each model. The logic of this analysis is as follows. Under a particular model, suppose you harvest at a fixed rate for a long period of time, allowing the population to reach an equilibrium size. You can calculate the annual harvest derived from that equilibrium with that harvest rate. Now, consider all possible fixed harvest rates, and choose the one that results in the highest annual harvest. That's the "optimal equilibrium point," which is the point where harvest is maximized, and the point that the state-dependent optimal policies seek to move toward.

The decision whether to adjust the balance equation bias by correcting survival or recruitment rates does not have a large effect on the equilibrium dynamics (Fig. 7). Because the equilibrium dynamics drive the state-dependent optimal decision strategy, the method of correcting the bias also is not likely to strongly affect the decision policy. In the interest of parsimony, we decided to consider only the hybrid correction method, as described above. The four alternative models under consideration all correct the bias in this way.

The equilibrium population sizes as a function of adult male harvest rate are shown for the four alternative models (Fig. 8, Table 3). The four models differ substantially in their predictions regarding the potential carrying capacity. This result is a realistic expression of uncertainty, since we have never observed what the mid-continent mallard population would do in the absence of harvest. The four models have relatively similar optimal equilibrium points (just under 6 million for three of the models, and near 9 million for the fourth), but require substantially different harvest rates to achieve those equilibria (ranging from 9% to 28%). Thus, these four models, while all quite plausible given our current understanding of mallard population dynamics, capture a range of uncertainty about management implications.

Optimal State-Dependent Policies

We derived optimal harvest strategies for each of the four models using stochastic dynamic programming (Lubow 1995), conditioning on the current set of regulatory alternatives and the current objective function: maximizing long-term cumulative harvest subject to a devaluation of harvest when the population falls below the North American goal of 8.8 million. (Note that this goal was increased from 8.7 million to reflect the 1998 revision of the North American Waterfowl Management Plan.) We also simulated these policies for long time periods to understand their expected dynamics.

Under the compensatory models (ScRs and ScRw, Figs. 9A and 9B), the optimal policy is to have a liberal season (current estimate of harvest rate = 0.12), regardless of population size or number of ponds, because at any harvest level less than 20%, harvest has no effect on the population size. Under the strongly density-dependent model (ScRs), the density-dependence regulates the population and keeps it within narrow bounds. Under the weakly density-dependent model (ScRw), the density-dependence does not exert as strong a regulatory effect, and the population size fluctuates greatly.

To understand the optimal policies for the additive models (SaRs and SaRw), it's necessary to first consider what the optimal policies would be if the North American goal were not used to devalue harvest (Figs. 9E and 9F). For the strongly density-dependent model (SaRs), the optimal policy is to always use liberal regulations, resulting in a mean population size of 6.7 million (Table 4). This is higher than the optimal equilibrium point of 6.0 million (Table 3), because the optimal harvest rate (14.8%) cannot be achieved with the liberal package. At low population sizes, the optimal strategy is "knife-edged" (i.e., it jumps from a closed season to a liberal package over a narrow range of BPOP), for the following two reasons: (1) the large amount of overlap in the harvest rates under the various packages; and (2) the benefit of moving the population size back toward the equilibrium point as quickly as possible. For the weakly density-dependent model (SaRw, Fig. 9F), the optimal policy is much more conservative than under the other models, because this model results in a lower potential for population growth. The optimal harvest rate (Table 3) is 9.1%, which is lower than the expected harvest rate under both the moderate and liberal packages. The mean population size under the optimal state-dependent policy, 8.1 million, is close to the optimal equilibrium population size of 8.7 million. Because of the low growth potential under this model, if the population size drifts below about 7 million, the optimal policy calls for closing the season in order to allow the population to return to higher levels.

When the North American goal is included in the objective function for the additive models, the optimal policies become more conservative (Figs. 9C and 9D). The reason is that the optimal equilibrium points for both of these models (SaRs and SaRw) are considerably below the North American goal. So, the optimal policy needs to seek a balance between maximizing harvest, yet avoiding having that harvest devalued too much. The more conservative strategies increase the mean population size (for SaRs, from 6.7 million to 7.1 million; and for SaRw, from 8.1 million to 9.2 million). This shift to a more conservative strategy is reflected in the expected frequency of each regulatory package (Table 4).

MODEL WEIGHT UPDATING

Model weights were calculated as Bayesian probabilities, which reflect the cumulative ability of the individual alternative models to predict observed changes in population size. The Bayesian probability for each model is a function of the model's previous (or prior) weight and the likelihood of the observed population size under the model. We used Bayes' theorem to calculate model weights from a comparison of predicted and observed population sizes for the years 1996-2001, starting with equal model weights in 1995. For the purposes of updating, we predicted the BPOP in the traditional survey area (reference areas 1-7, 12, and 13) in year $t + 1$, from the traditional BPOP, May ponds, and adult male harvest rate in year t.

For 5 of the past 6 years, the likelihoods of the four models have been quite similar, and thus have provided little evidence favoring any models (Table 5). Further, in 4 of the past 6 years, the observed BPOP has been within the range of predictions from the four models. Thus, collectively the models have done a good job of prediction. In 1999, however, the observed BPOP was considerably higher than the expected value under any of the models (although it was not unreasonable with regard to the predicted distributions). The effect of this one year was to

favor those models that tended to predict higher population sizes, namely, ScRw and SaRw, and the collective weight associated with these two models increased from 55.3% to 85.3%. The current (2001) model weights suggest the weakly density-dependent model is favored 86.3% to 13.7% over the strongly density-dependent model; and there is roughly equal evidence for the compensatory (45.7%) and additive (54.3%) models. For comparison, under the old AHM protocol (balance equation, submodels, and variances), only one model (SaRs) would have any significant weight associated with it in 2001 (Table 5).

Under the optimal harvest strategy associated with the set of four models and their 2001 weights (and including the North American goal), the liberal alternative is expected to be chosen 52.5% of the time, with closed seasons 22.3% of the time, and a small portion of time spent in the other packages (Table 4, Fig. 10). The population size would be expected to average 7.2 million.

FUTURE WORK

The AHM Working Group, in conjunction with colleagues in federal, state, and private institutions, has continuously scrutinized the models and methods used to generate harvest regulations through the AHM process. The set of revisions described herein represents the culmination of more than 2 years of analysis and careful deliberation, a process that is annually documented in the AHM reports (available online at http://migratorybirds.fws.gov/reports/reports.html).

We expect that there will be additional improvements made in the AHM models and methods in the future, as new data, analyses, methods, and insights become available. While the AHM Working Group has the primary responsibility for pursuing such technical improvements, the process is open to, and is enhanced by, contributions from other scientists.

We recognize a number of areas where future work is needed to improve the AHM models. Some of this work is on-going and is documented in previous AHM reports, but was beyond the scope of the revisions we could include this year. (1) *Improving recruitment sub-models*. Efforts are underway to develop better models of recruitment to reflect uncertainty about the functional form of density-dependence, and to capture large-scale landscape changes using an expanded set of predictors (including the distribution of ponds in both the U.S. and Canada). (2) *Improving survival sub-models*. We are continuing to develop a more mechanistic model for the compensatory hypothesis. (3) *Understanding the cause of the balance equation bias*. In the work described in this report, we used an empirical correction for the bias in the balance equation. We intend to continue to look for patterns in this bias, and track it on an annual basis. In addition, there is an external study that is investigating whether there is any bias in the age-ratio data. (4) *Improving the updating procedure*. Currently, only information about changes in the population size is used to adjust model weights. We are exploring whether we can also use information about observed annual survival rates and age-ratios to inform the model weight updating. (5) *Improving estimates of harvest rate*. The new reward-band study will provide better estimates of harvest rates, which will be incorporated into the AHM mallard models annually.

LITERATURE CITED

Anderson, D.R. and K.P. Burnham. 1976. Population ecology of the mallard. VI. The effect of exploitation on survival. U.S. Fish and Wildlife Service Resource Publication 128.

Anderson, D.R, and C.J. Henny. 1972. Population ecology of the mallard. I. A review of previous studies and the distribution and migration from breeding areas. U.S. Fish and Wildlife Service Resource Publication 105.

Brownie, C., D.R. Anderson, K.P. Burnham, and D.S. Robson. 1985. Statistical inference from band recovery data—a handbook, 2nd edition. U. S. Fish and Wildlife Service Resource Publication 156.

Draper, N.R. and H. Smith. 1981. Applied regression analysis. Second ed. John Wiley and Sons, New York, New York.

Johnson, F.A., C.T. Moore, W.L. Kendall, J.A. Dubovsky, D.F. Caithamer, J.R. Kelley, Jr., and B.K. Williams. 1997. Uncertainty and the management of mallard harvests. Journal of Wildlife Management 61(1):202-216.

Johnson, F.A., B.K. Williams, J.D. Nichols, J.E. Hines, W.L. Kendall, G.W. Smith, and D.F. Caithamer. 1993. Developing an adaptive management strategy for harvesting waterfowl in North America. Transactions of the North American Wildlife and Natural Resources Conference 58:565-583.

Lubow, B.C. 1995. SDP: Generalized software for solving stochastic dynamic optimization problems. Wildlife Society Bulletin 23:738-742.

Munro, R.E., and C.F. Kimball. 1982. Population ecology of the mallard. VII. Distribution and derivation of the harvest. U.S. Fish and Wildlife Resource Publication 147.

Nichols, J.D., R.J. Blohm, R.E. Reynolds, R.E. Trost, J.E. Hines, and J.P. Bladen. 1991. Band reporting rates for mallards with reward bands of different dollar values. Journal of Wildlife Management 55:119-126.

Nichols, J.D., R.E. Reynolds, R.J. Blohm, R.E. Trost, J.E. Hines, and J.P. Bladen. 1995. Geographic variation in band reporting rates for mallards based on reward banding. Journal of Wildlife Management 59:697-708.

Runge, M.C. and F.A Johnson. 2002. The importance of functional form in optimal control solutions of problems in population dynamics. Ecology 83:1357-1371.

Smith, G.W. and R.E. Reynolds. 1992. Hunting and mallard survival, 1979-1988. Journal of Wildlife Management 56:306-36.

Table 1. Correlation between observed annual changes in BPOP and predicted changes in BPOP, using the balance equation and various data sets.

Combination	Age-ratio Data Set	Survival Data Set	Correlation
1	Fall M1 (female)	Pooled	0.383
2	Fall M1 (female)	Weighted	0.464
3	Fall M2 (female)	Pooled	0.556
4	Fall M2 (female)	Weighted	0.637
5	Fall M3 (female)	Pooled	0.575
6	Fall M3 (female)	Weighted	0.657
7	Harvest (female)	Pooled	0.593
**8**	**Harvest (female)**	**Weighted**	**0.671**
9	Harvest (male)	Pooled	0.533
10	Harvest (male)	Weighted	0.608

Table 2. Linear regression of female fall age-ratio on Canadian ponds (`Ponds`) and breeding population size (`BPOP`), 1961-1995. Note that `Ponds` and `BPOP` were both expressed in millions.

```
The regression equation is
R(t) = 0.928 + 0.123 Ponds(t) - 0.0752 BPOP(t)

Predictor         Coef        StDev           T        P
Constant        0.9278       0.1126        8.24    0.000
Ponds(t)       0.12293      0.01803        6.82    0.000
BPOP(t)       -0.07518      0.01710       -4.40    0.000

S = 0.1149      R-Sq = 61.3%     R-Sq(adj) = 58.9%

Analysis of Variance
Source           DF          SS          MS         F        P
Regression        2     0.66868     0.33434     25.33    0.000
Residual Error   32     0.42237     0.01320
Total            34     1.09105
```

Table 3. Carrying capacity and optimal equilibrium characteristics for the four alternative models. The population sizes and total harvest are expressed in millions, and include the traditional survey areas and the Lake States. The carrying capacity is the equilibrium population size in the absence of harvest. The optimal equilibrium point (N_{eq}^*) is the population size that provides the greatest annual harvest (H_{eq}^*) when subjected to the optimal harvest rate (h^*).

Model	Carrying Capacity	N_{eq}^*	h^*	H_{eq}^*
ScRs	7.07	5.58	0.282	1.57
ScRw	5.77	5.75	0.184	1.06
SaRs	10.50	6.00	0.148	0.89
SaRw	16.17	8.72	0.091	0.79

Table 4. Expected performance characteristics of optimal, model-specific harvest strategies for mid-continent mallards. Each optimal policy was simulated, under the model used to generate it, for 500 years. The statistics shown were calculated from the last 400 years of the simulation.

	BPOP		Expected frequency of regulations				
**Model**	**Mean**	**Variance**	**C**	**VR**	**R**	**M**	**L**
ScRs	6.83	2.33	0	0	0	0	100
ScRw	5.23	6.73	0	0	0	0	100
SaRs, no NA goal	6.70	2.27	0	0	0	0	100
SaRs, NA goal	7.08	1.70	0.8	3.8	9.8	16.5	69.3
SaRw, no NA goal	8.11	2.27	15.8	8.8	14.3	9.3	52.0
SaRw, NA goal	9.15	2.34	19.3	15.5	17.0	16.0	32.3
2001	7.22	2.61	22.3	8.8	11.8	4.8	52.5

Table 5. Model-specific probabilities of breeding population size (in millions), observed population size, and resulting likelihoods and model weights for each model of mid-continent mallard population dynamics. Model weights were assumed to be equal in 1995. Note that the BPOPs (both predicted and observed) are for the traditional survey area only.

**Year**	**Parameter**	**ScRs**	**ScRw**	**SaRs**	**SaRw**	**BPOP (observed)**
1996	BPOP (predicted)	7.67	8.02	7.70	8.03	7.94
	Likelihood	2.50	2.56	2.51	2.55	
	Weight	0.247	0.253	0.248	0.252	
1997	BPOP (predicted)	8.06	8.18	8.08	8.19	9.94
	Likelihood	1.03	1.17	1.05	1.18	
	Weight	0.230	0.266	0.235	0.269	
1998	BPOP (predicted)	9.10	9.93	8.98	9.75	9.64
	Likelihood	2.39	2.52	2.31	2.55	
	Weight	0.224	0.273	0.222	0.280	
1999	BPOP (predicted)	7.43	8.47	7.63	8.62	10.81
	Likelihood	0.14	0.75	0.21	0.90	
	Weight	0.060	0.383	0.087	0.470	
2000	BPOP (predicted)	8.59	9.99	8.94	10.32	9.47
	Likelihood	2.11	2.41	2.39	2.20	
	Weight	0.055	0.403	0.091	0.45	
2001	BPOP (predicted)	7.33	8.30	7.34	8.25	7.90
	Likelihood	2.27	2.44	2.29	2.46	
	Weight	0.052	0.405	0.086	0.457	
2001	Weights (under the old protocol)	0.000	0.000	0.975	0.025	

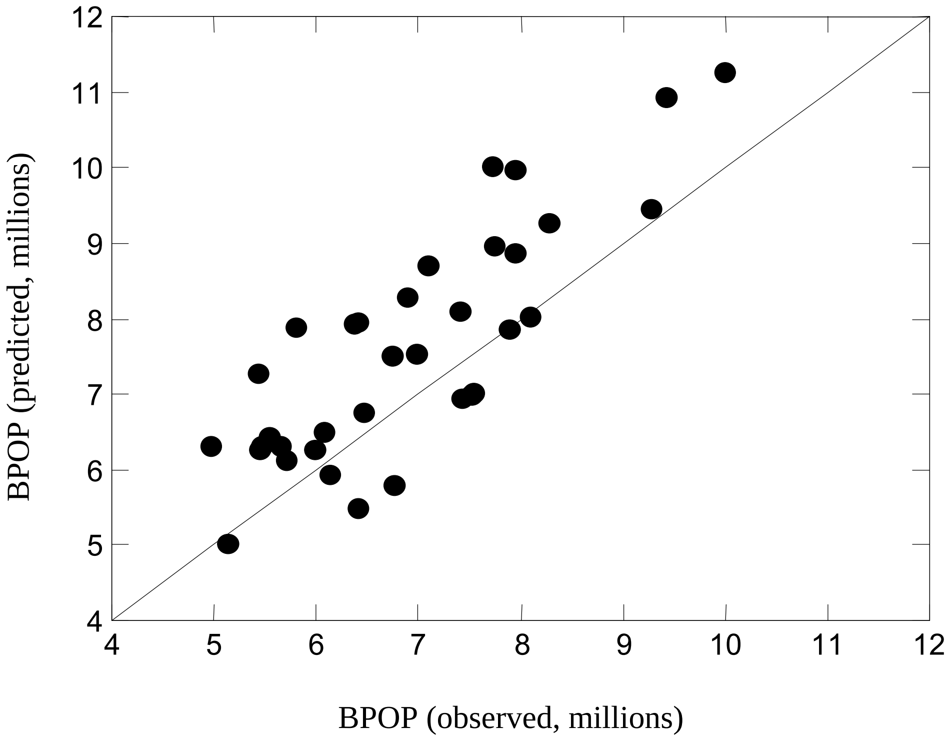

Fig. 1. Predicted versus observed breeding population size for the traditional mid-continent mallard region, 1962-1996. The predictions are generated using the balance equation, uncorrected for bias.

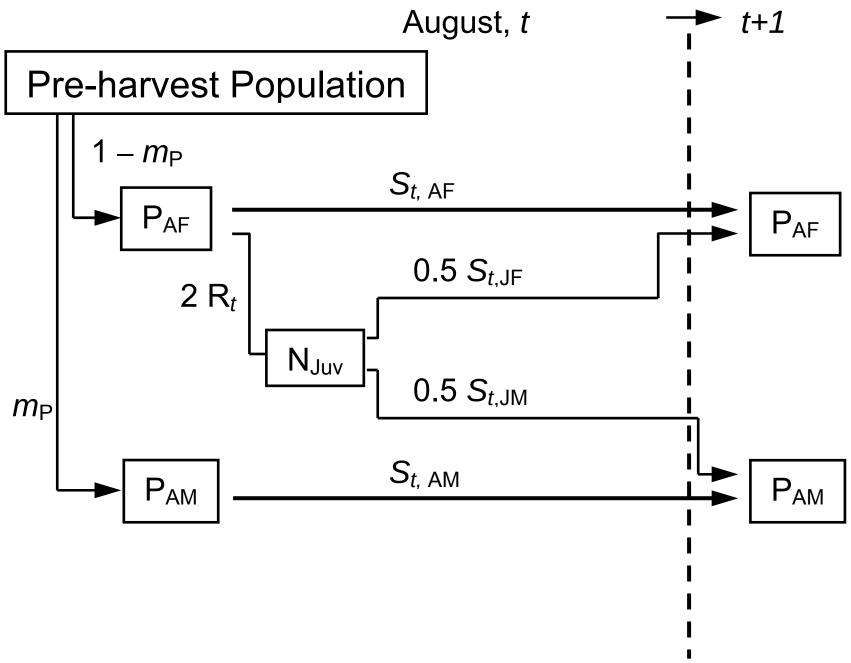

Fig. 2. Schematic of August-to-August balance equation.

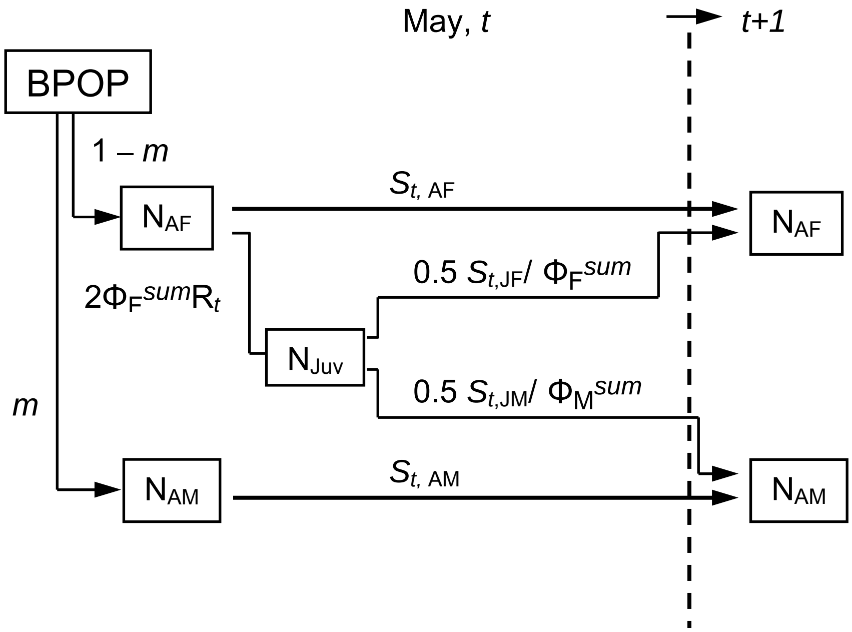

Fig. 3. Schematic of May-to-May balance equation.

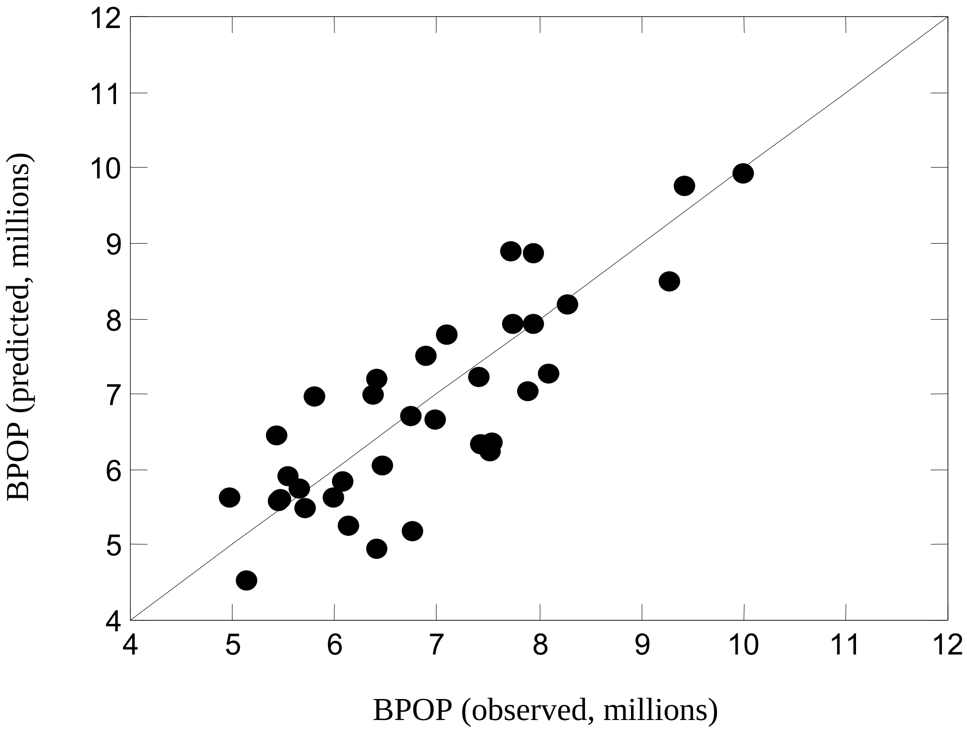

Fig. 4. Predicted versus observed breeding population size for the traditional mid-continent mallard region, 1962-1996, using the hybrid bias-correction in the balance equation to generate the predictions.

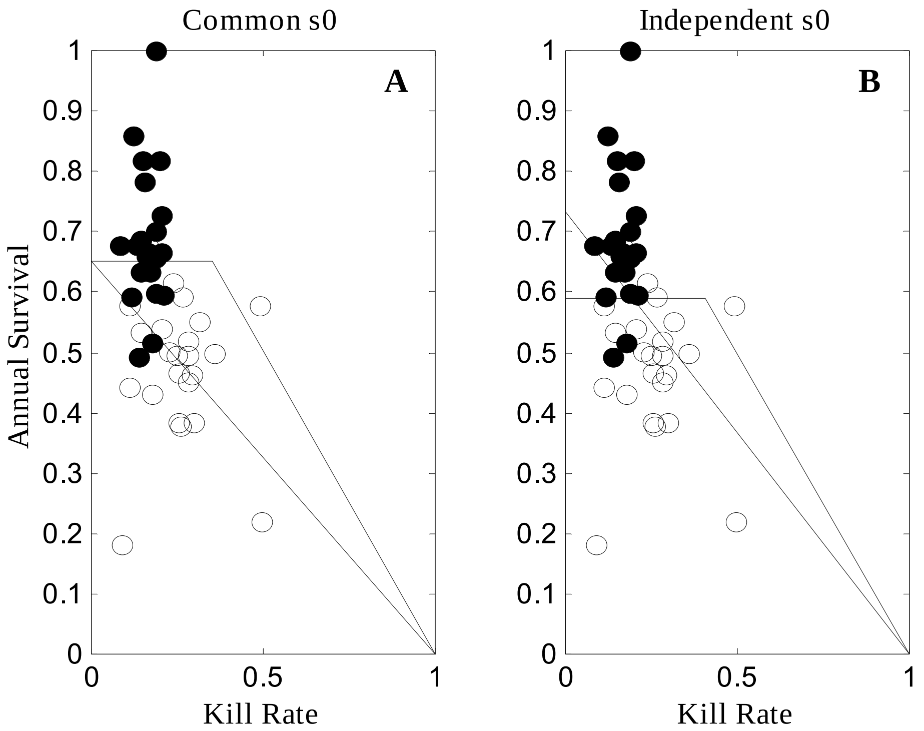

Fig. 5. Annual survival rates versus kill rates for mid-continent male mallards banded in reference area 2 during 1961-1986. The solid circles depict juvenile males, the open circles adult males. (A) Additive and compensatory models fit to data using a common value for survival in the absence of harvest. (B) Additive and compensatory models fit independently to data.

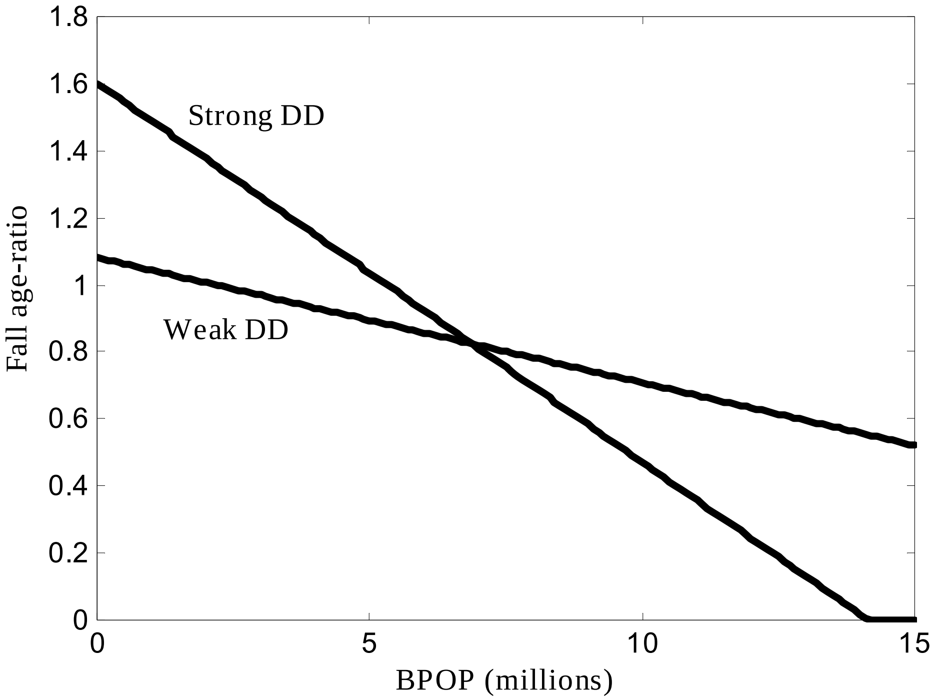

Fig. 6. Predicted fall age-ratio versus breeding population size for the two alternative recruitment models. For both curves, the mean value for Canadian ponds (3.36) was used.

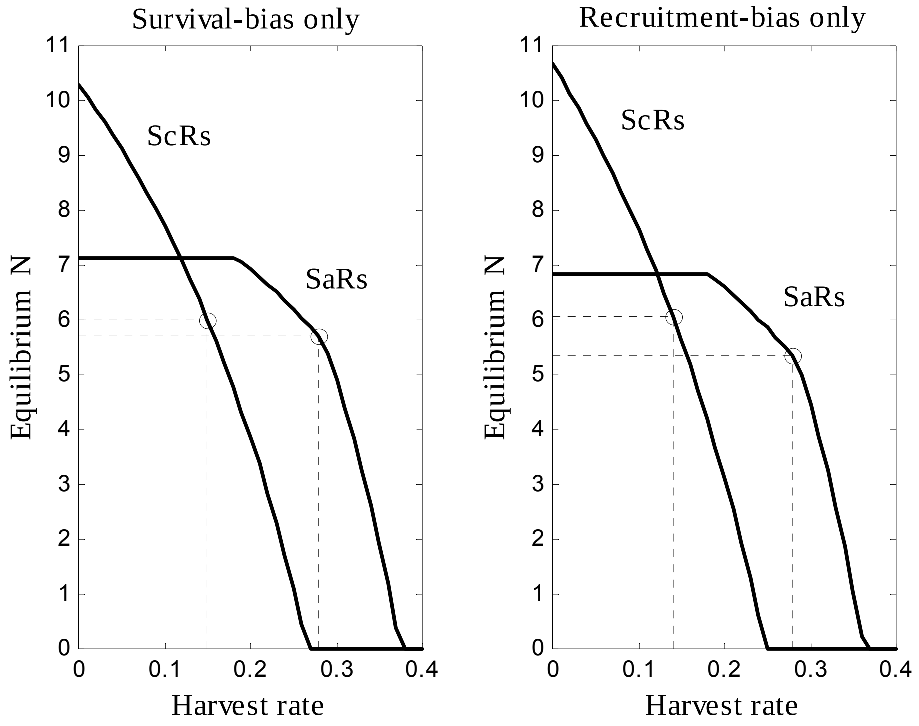

Fig. 7. Equilibrium curves for the strong density-dependent recruitment models: comparison of survival and recruitment bias corrections. The solid lines show the equilibrium population size (BPOP, in millions) as a function of a fixed harvest rate (adult male). The open circles and dashed lines locate the optimal equilibrium points (where annual harvest is maximized).

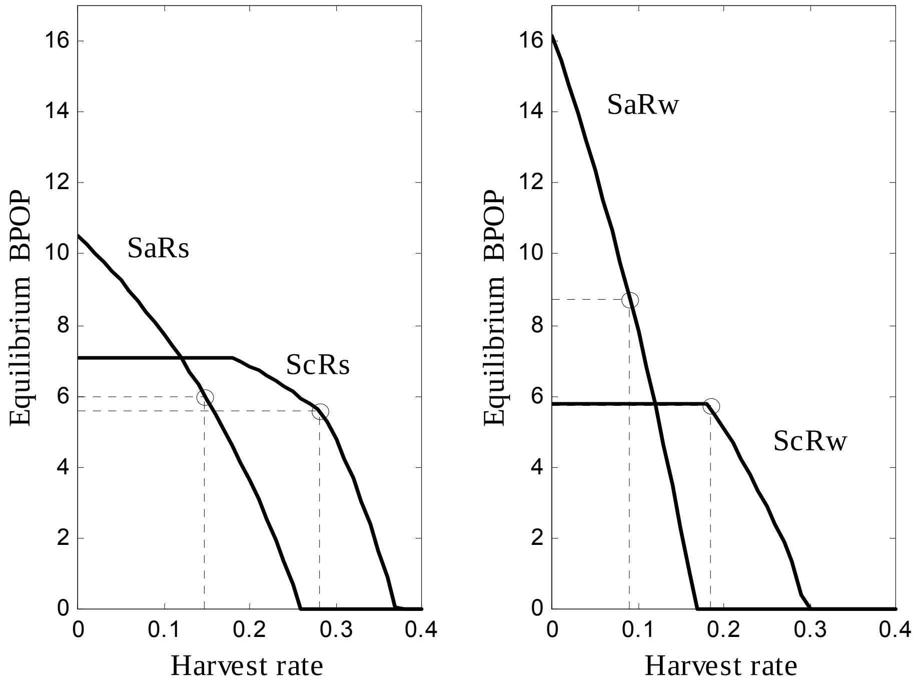

Fig. 8. Equilibrium curves for the four alternative models, all using the hybrid bias correction. See Fig. 7 for legend.

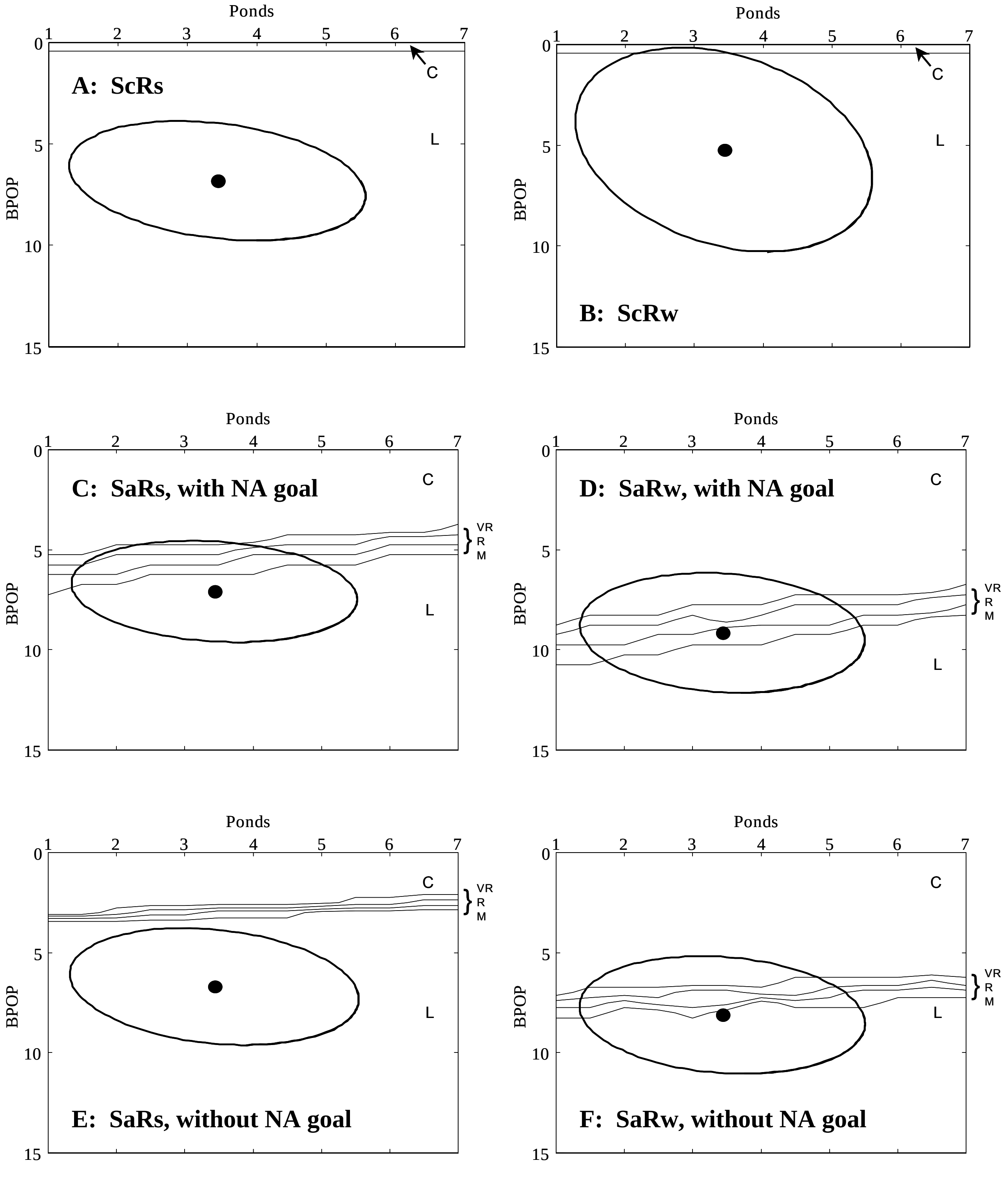

Fig. 9. State-dependent optimal policies for the individual models. The two state variables, BPOP (including Lake States, in millions) and Canadian Ponds (in millions) compose the axes. Contours indicate the transitions between regulation packages. The solid circle shows the simulated mean value of the state variables. The ellipse indicates the region where 95% of the simulated observations occur. (A-D) The policies for the four alternative models. (E-F) The policies for the additive models, with the North American goal removed from the objective function.

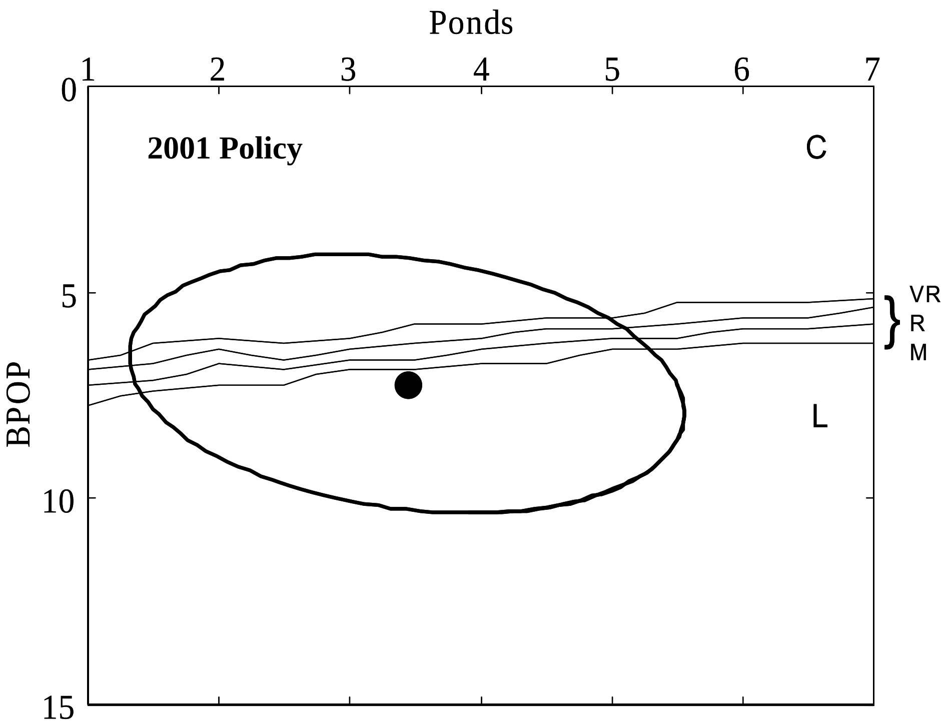

Fig. 10. State-dependent optimal policy for the 2001 model. See Fig. 9 for legend. This model averages the four alternative models according to their 2001 weights.
